# TAB2 deficiency induces dilated cardiomyopathy by promoting mitochondrial calcium overload in human iPSC-derived cardiomyocytes

**DOI:** 10.1186/s10020-025-01103-x

**Published:** 2025-02-04

**Authors:** Wenrui Sun, Jianchao Zhang, Shuang Li, Wanrong Fu, Yangyang Liu, Mengduan Liu, Jianzeng Dong, Xiaoyan Zhao, Xiaowei Li

**Affiliations:** 1 Department of Cardiology, The First Affiliated Hospital of Zhengzhou University, Zhengzhou University, Longhu Zhonghuan Road No. 1, Jinshui District, Zhengzhou, 450052 China; 2 Henan Key Laboratory of Hereditary Cardiovascular Diseases, Zhengzhou, 450052 China; 3 School of Life Sciences, Zhengzhou University, Zhengzhou, 450001 China; 4 Department of Cardiology, Beijing Anzhen Hospital, Capital Medical University, National Clinical Research Centre for Cardiovascular Diseases, No. 2 Beijing Anzhen Road, Chaoyang District, Beijing, 100029 China

**Keywords:** TAB2 knockout, IPSCs, Dilated cardiomyopathy, Mitochondrial calcium overload

## Abstract

**Background:**

TGF-β-activated kinase 1 binding protein 2 (TAB2) is an intermediary protein that links Tumor necrosis factor receptor 1 (TNFR1) and other receptor signals to the TGF-β-activated kinase 1 (TAK1) signaling complex. TAB2 frameshift mutations have been linked to dilated cardiomyopathy (DCM), while the exact mechanism needs further investigation.

**Methods:**

In this study, we generated a TAB2 compound heterozygous knockout cell line in induced pluripotent stem cells (iPSCs) derived from a healthy individual using CRISPR/Cas9 technology. IPSCs are not species-dependent, are readily accessible, and raise fewer ethical concerns.

**Results:**

TAB2 disruption had no impact on the cardiac differentiation of iPSCs and led to confirmed TAB2 deficiency in human iPSC-derived cardiomyocytes (hiPSC-CMs). TAB2-deficient hiPSC-CMs were found to develop phenotypic features of DCM, such as distorted sarcomeric ultrastructure, decreased contractility and energy production, and mitochondrial damage at day 30 post differentiation. Paradoxically, TAB2 knockout cell lines showed abnormal calcium handling after 40 days, later than reduced contractility, suggesting that the main cause of impaired contractility was abnormal energy production due to mitochondrial damage. As early as day 25, TAB2 knockout cardiomyocytes showed significant mitochondrial calcium overload, which can lead to mitochondrial damage. Furthermore, TAB2 knockout activated receptor-interacting protein kinase 1 (RIPK1), leading to an increase in mitochondrial calcium uniporter (MCU) expression, thereby augmenting the uptake of mitochondrial calcium ions. Finally, the application of the RIPK1 inhibitor Nec-1s prevents the progression of these phenotypes.

**Conclusions:**

In summary, TAB2 abatement cardiomyocytes mimic dilated cardiomyopathy in vitro. This finding emphasizes the importance of using a human model to study the underlying mechanisms of this specific disease. More importantly, the discovery of a unique pathogenic pathway introduces a new notion for the future management of dilated cardiomyopathy.

**Supplementary Information:**

The online version contains supplementary material available at 10.1186/s10020-025-01103-x.

## Background

Dilated cardiomyopathy is characterized by left ventricular or biventricular dilation and impaired contraction, which cannot be explained by abnormal loading conditions (such as hypertension and valvular heart disease) or coronary artery disease, and has been diagnosed as a clinical entity (McKenna et al. 2017; Schultheiss et al. 2019). As the condition progresses, it leads to heart failure and arrhythmias, posing serious health risks to individuals. Hershberger, Ray E and colleagues have proposed a prevalence ratio of 1:250 for idiopathic DCM based on the relationship between idiopathic DCM and hypertrophic cardiomyopathy (Hershberger et al. 2013). To date, over 50 mutations in genes encoding structural proteins such as sarcomeres, cytoskeleton, ion channels, and so on, have been identified to contribute to DCM (Merlo et al. 2018). Furthermore, mutations in regulatory protein genes that influence the onset and progression of DCM, such as TAB2, are continuously being investigated.

TAB2 is a vector protein that connects TNFR1 and other receptors to TAK1 signal transduction complex (Takaesu et al. 2000), a gene located on chromosome 6q25.1. TAK1, a member of the MAPK kinase (MAPKKK) family, is involved in the regulation of various physiological and pathological processes and is a key regulator of cell death and necrotic apoptosis (Geng et al. 2017). Pro-inflammatory cytokines such as TNF-α and IL-1β activate TAK1, which in turn mediates the activation of nuclear factor κB (NF-κB), c-Jun N-terminal kinase (JNK), and p38 through TABs. Among them, TAB1 is responsible for the early activation of the downstream pathway, while TAB2 and TAB3 are responsible for continuous activation of the pathway (Xu and Lei 2020). TAB2 plays a critical role in pathway activation, and its deficiency can lead to pathophysiological changes. Various studies have reported cases in which nonsense, or small insertion/deletion mutations of the TAB2 gene result in congenital heart disease and dilated cardiomyopathy (Chen et al. 2020; Engwerda et al. 2021; Koene et al. 2022; Vasilescu et al. 2018). However, some TAB2 mutations are associated only with dilated cardiomyopathy, without causing congenital heart disease (Caulfield et al. 2018; Cheng et al. 2017; Engwerda et al. 2021; Vasilescu et al. 2018). The mechanism remains unclear.

**Table 1:** Primer sequences used for q-PCR

Gene	Forward 5′–3′	Reverse 5′–3′
*GAPDH*	GGAGCGAGATCCCTCCAAAAT	GGCTGTTGTCATACTTCTCATGG
*NPPA*	ACAATGCCGTGTCCAACGCAGA	CTTCATTCGGCTCACTGAGCAC
*NPPB*	TCTGGCTGCTTTGGGAGGAAGA	CCTTGTGGAATCAGAAGCAGGTG
*ND1*	GGCTATATACAACTACGCAAAGGC	GGTAGATGTGGCGGGTTTTAGG
*ND2*	CCATCTTTGCAGGCACACTCATCA	ATTATGGATGCGGTTGCTTGCGTG
*ACTB*	CATGTACGTTGCTATCCAGGC	CTCCTTAATGTCACGCACGAT
*REX1*	GCCTTATGTGATGGCTATGTGT	ACCCCTTATGACGCATTCTATGT
*SOX2*	GCTACAGCATGATGCAGGACCA	TCTGCGAGCTGGTCATGGAGTT
*NANOG*	CTCCAACATCCTGAACCTCAGC	CGTCACACCATTGCTATTCTTCG
*MVP*	TTAGACCAGAGCTGCTCAGA	CTCACTGCAACGTCCACCTA
*ATP8B4*	ATCAGCAGTTAGTTGAGCCG	CTGAGTTTAGAGAAGGCACT
*TEKT5*	ATTGGGAGATGAGGAGTGGT	GGAGTGGAATAGCTGGATCA
*CRISPR/Cas9 plasmid*	GCCGTGATCACCGACGAGTA	CGTCTGCTCTTGCTCAGTCT
*Mycoplasma 16srRNA*	GGGAGCAAACAGGATTAGATACCCT	TGCACCATCTGTCACTCTGTTAACCTC
*TAB2*	TCAGCACCACATCTTGGATT	ATTGGCATGTAGACATGAGAG
*ATP2A2*	GGACTTTGAAGGCGTGGATTGTG	CTCAGCAAGGACTGGTTTTCGG
*RYR2*	AGAACTTACACACGCGACCTG	CATCTCTAACCGGACCATACTGC
*CACNA1C*	CAGAGGCTACGATTTGAGGA	GCTTCACAAAGAGGTCGTGT
*RIPK1*	TATCCCAGTGCCTGAGACCAAC	GTAGGCTCCAATCTGAATGCCAG
*MCU*	CAGCACTGTTGTGCCCTCTGAT	GGCTTGAGTGTGAACTGACAGC
*gRNA*	ccgGTCTGTCGAGTTGTACCACC	aacGGTGGTACAACTCGACAGAC

Due to most of the DCM caused by TAB2 mutations are nonsense and frameshift mutations, with a few being missense mutations. We used CRISPR/Cas9 technology to create a TAB2 knockout cardiomyocyte model from healthy human iPSCs. This model enables us to investigate the mechanisms through which TAB2 knockout contributes to dilated cardiomyopathy (DCM) and heart failure (HF). Our study revealed that TAB2 knockout in cardiomyocytes results in increased expression of receptor interacting protein kinase 1 (RIPK1) and mitochondrial calcium uniporter (MCU). Consequently, this leads to mitochondrial calcium overload, damage, and ultimately the development of DCM. Notably, we observed that treatment with the RIPK1 inhibitor Nec-1s significantly mitigates these detrimental effects, suggesting a potential therapeutic strategy for DCM.

## Methods

### Cell culture and cardiac differentiation

Wild-type (WT) iPSCs were obtained from the ZZUNEUi011-A cell line, which was previously established from peripheral blood mononuclear cells (PBMCs) of a healthy 27-year-old female individual (Fu et al. 2021). The WT and KO hiPSC lines were cultured on matrigel and the fluid was replaced daily using mTeSR in 5% CO2, 37 ℃ environment. When the cell confluence reached 90–100%, the cardiomyocyte differentiation medium was replaced and purified using the lactic acid metabolism method, as we mentioned before (Li et al. 2019).

### Genome editing

The sgRNA targeting for exon 4 of TAB2 (GTCTGTCGAGTTGTACCACC) was designed by the CRISPR design tool (http://crispr.mit.edu/). The gRNA and epiCRISPR vector were electroporated into hiPSCs used CUY21EDITII (BEX) system. Transfected cells were inoculated into a 6-well plate coated with matrix gel, and 10 µM Rho kinase inhibitor Y-27632 was added for the first 24 h of culture. On the third day of culturing, puromycin was added for drug screening, and the surviving clones were selected and transferred to 24-well plates for PCR detection.

To investigate the calcium handling, we generated WT-GCaMP and TAB2-KO-GCaMP hiPSC as previously described (Li et al. 2019). Briefly, the generation of hiPSC-GCaMP and TAB2^−/−^ GCaMP is as follows: 2.5 μg AAVS1_sgRNA plasmid (Addgene, #100554, USA) and 2.5 μg pAAVS1-PC-GCaMP6f plasmid (Addgene, #73503) were electroporated together into hiPSC and TAB2^−/−^hiPSCs. Purinomycin drug screening and identification of clones can be conducted in the manner described above.

### Immunostaining and imaging analyses

The cells were fixed with 4% paraformaldehyde (Sigma, USA) for 30 min, permeabilized with 0.5% Triton X-100 (Sigma) for 15 min, and blocked with BSA for 30 min. The cells were then incubated overnight with the primary antibody cTnT (1:100, ab209813, Abcam, USA) and α-actinin (1:100, ab137346, Abcam, USA) at 4 °C, washed with PBS, and the diluted secondary antibody (Alexa Fluor 594 goat anti-Mouse IgG, A11032, 1:200; Alexa Fluor 488 goat anti-Rabbit IgG, A11034, 1:200, Invitrogen, USA) was incubated at room temperature for 2 h. After being washed again with PBS, the cells were stained with DAPI (300 nM, Invitrogen, USA) at room temperature for 5 min. Confocal fluorescence microscopy was used for observation and photography.

### Flow cytometry

The cardiomyocytes were digested with digestive enzymes, washed three times with PBS and fixed with 4% formaldehyde for half an hour. Then the primary antibody was incubated for half an hour and cleaned with PBS, including SSEA4 (1:200, Cat#41–4000, Thermo Fisher Scientific, USA) and cTnT (1:200, ab209813, Abcam, USA) and the secondary antibody (Alexa Fluor 488 goat anti-Rabbit IgG, A11034, 1:200; Alexa Fluor 488 goat anti-Mouse IgG, A1102, 1:200, Invitrogen, USA) was incubated again for half an hour and cleaned with PBS.

Quantification of mitochondria, total reactive oxygen species (ROS) and mitochondrial calcium: The digested cardiomyocytes were stained with Mitotracker Green, DCFH-DA and Rhod-2, AM probe. The cells to be detected were removed from the cell medium, cleaned with PBS, and incubated with the mentioned dyes for 20–30 min each. The working fluid was removed, and the cardiomyocytes were rinsed with PBS three times before digestion. The samples are placed in EP tubes on ice for flow detection. The results were analyzed using FlowJo software. The average fluorescence intensity was used as a metric to compare the mitochondrial content, total ROS content, and mitochondrial calcium content of wild-type (WT) and KO cells.

### Ca^2+^ imaging

Measurement of calcium transients, which we have described previously (Guo et al. 2024). In simple terms, TAB2-KO-GCaMP hiPSC-CMs were seeded onto confocal dishes. Intracellular Ca^2+^ flux was imaged using the Olympus cardiomyocyte contractility and calcium ion synchronous measurement system. Combined and inverted fluorescence microscopy. To detect the release of calcium ions triggered by caffeine, a concentration of 10 mM caffeine was used to stimulate the sarcoplasmic reticulum for Ca^2+^ release. The results were analyzed using ImageJ software.

### Detection of myocardial contractility

Plexithermo HCell series single myocardial cell function detection system was used to measure the force of myocardial contractility (Zhang et al. 2024). In a nutshell, cardiomyocytes were inoculated into a 6-well hydrogel culture plate (PLEXITHERMO) and observed under an inverted fluorescence microscope. The contractile activity of the cells was recorded using the Olympus cardiomyocyte contractile force and calcium ion synchronous measurement system. The above videos were analyzed using AUTO-C software to determine the parameters of cell contractile force.

### RNA extraction and quantitative real-time PCR (qRT-PCR)

Total RNA was isolated using TRIzol reagent (Life Technologies) according to the manufacturer’s procedure, and then reverse-transcribed into CDNA using PrimeScript™ RT Master Mix (Takara). QRT-PCR was done on QuantStudio 3 (Thermo) with 2 SYBR Master Mix (Takara). The relative quantification of targeted genes is computed using the 2-ΔΔCT approach. Table1 displays the primer sequence.

### Western blots

Total protein was isolated from RIPA lysate including a protease inhibitor cocktail and phosphatase inhibitor cocktail. The protein concentration was determined using a BCA kit, then 4*loading buffer and DTT were added for boiling. The protein was isolated on a 6–10% sodium dodecyl sulfate poly-acrylamide gels based on its molecular weight and transferred to a PVDF membrane. The membrane was then sealed with 5% skim milk before the primary antibody was incubated overnight, including TAB2 (1:1000, 14410-1-AP, Proteintech, China), RIPK (1:1000, 17519-1-AP, Proteintech, China), MCU (1:1000, ab272488, Abcam, USA), SERCA2 (1:1000, #9580, Cell signaling Technology, USA), P-AMPK (1:1000, ab39400, Abcam, USA), P-CaMKII (1:1000, #12716, Cell Signaling Technology, USA) and GAPDH (1:1000, 60004-1-lg, Proteintech, CHINA). The second antibody (HRP-conjugated Affinipure goat Anti-Mouse IgG H&L (HRP), SA00001-1, 1:2000; HRP-conjugated Affinipure goat Anti-Rabbit IgG H&L (HRP), SA00001-2, 1:2000, Proteintech, CHINA) was incubated at room temperature for 2 h on the second day. After three more TBST cleanings, chemiluminescence can be done.

### RNA sequencing (RNA‑Seq) assay

The hiPSC-CMs were subjected to total RNA extraction, as detailed above. We obtained the raw image data file through Illumina Hiseq™ (Catalog, Wuhan, HB, China) and converted it into the original sequencing sequence through CASAVA base recognition analysis. First, the raw data quality value and other information were counted, and the sequencing data quality of the sample was visually assessed using FastQC. Subsequently, Trimmomatic was used for data processing to obtain clean data. The reference genome was used as the basis sequence. HISAT2 was employed to compare the quality-controlled sequencing data with the reference genome, and RSeQC was used to analyze the results. In addition, we can estimate gene expression levels by counting sequencing sequences (reads) that are targeted to genomic regions or gene exons. Next, we utilized DESeq for analysis, setting the screening condition as qValue < 0.05 and the difference multiple |FoldChange| > 2 to identify significantly different genes. Finally, functional enrichment analysis using clusterProfiler.

### Detection of ATP content in cells

ATP lysate was added to the 6-well plate, and the supernatant was collected after cell lysis. The luminescence values of these cells were measured using the enhanced ATP assay kit (S0027) on the chemiluminescence apparatus. The concentration of BCA protein was determined, and the results were standardized.

### Fluorescence intensity detection of Mitotracker Green in cardiomyocytes

A 1 mM stock solution was prepared by dissolving Mitotracker Green powder in DMSO. The working solution was then made by diluting the stock with PBS at a 1:5000 ratio and incubating the solution at 37 °C for 20 min, protected from light. Cells were washed with PBS, treated with the working solution for 30 min, and then washed three times before imaging under a fluorescence microscope.

### Fluorescence intensity detection of CellROS Green in cardiomyocytes

The stock solution was diluted with PBS at a 1:1000 ratio to create the working solution. After washing the cells, the working solution was added and incubated at 37 °C for 30 min, followed by three PBS washes and fluorescence imaging.

### Fluorescence intensity detection of Rhod-2 AM in cardiomyocytes

The Rhod-2/AM powder was dissolved in DMSO to prepare a 5 mM stock solution, which was stored at −20 °C. The working solution was made by diluting the stock in HBSS (without Ca^2^⁺ and Mg^2^⁺) to a final concentration of 5 μM. Cells were incubated with the working solution for 15–60 min, washed with HBSS, and then incubated for an additional 20–30 min to ensure complete de-esterification. Cells were then observed under a fluorescence microscope.

### Data analysis and statistics

Data was represented as mean ± standard deviation (SD). Student’s *t* test was used to evaluate the statistical significance of the differences between two groups. One-way ANOVA or two-way ANOVA were conducted to compare differences between multiple groups. *P* values less than 0.05 were considered statistically significant by Student’s *t* test (* *P* < 0.05, ** *P* < 0.01, *** *P* < 0.001, **** *P* < 0.0001; ns, not significant).

## Results

### Generation of homozygous TAB2^−/−^ hiPSCs

**Fig. 1:**
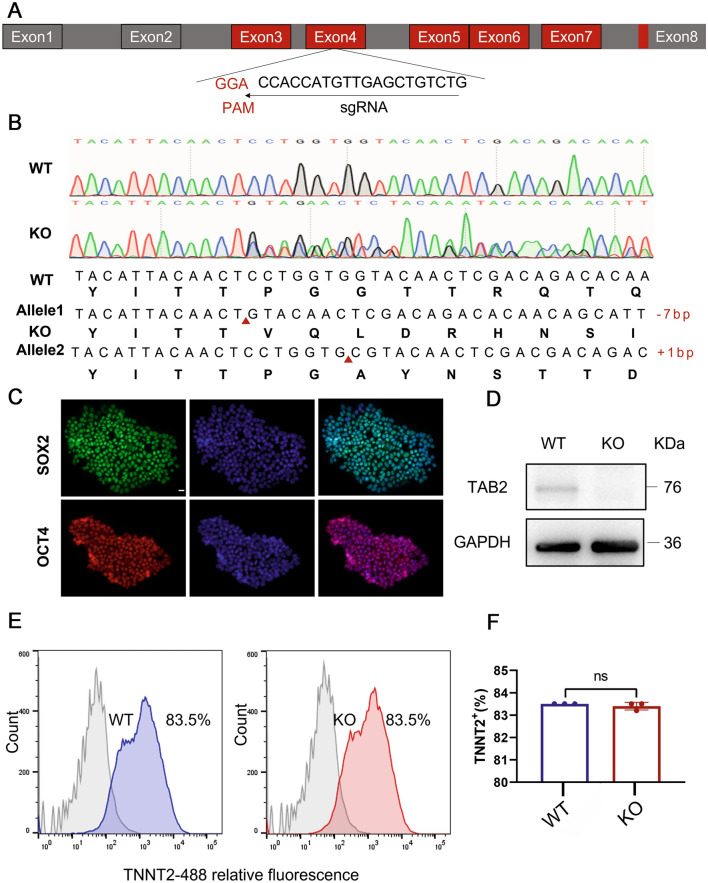
TAB2 knockout does not affect the pluripotency of hiPSCs and differentiation into hiPSC-CMs with CHIR99021 and Wnt-C59. **A** Structure of the TAB2 locus showing localization of gRNA for epiCRISPR/Cas9 editing. **B** Sequence chromatograms demonstrate a homozygous TAB2 gene KO line in which 1 allele deleted 7 nucleotides (CCTGGTG) and another allele inserted 1 nucleotide (**C**). **C** Immunofluorescence staining of TAB2-KO colonies for the pluripotency markers SOX2 and OCT4. Scale bar, 20 μm. **D** Immunoblot analysis of TAB2 in WT and TAB2 KO CMs at day 20 (*n* = 3). **E**,**F** Flow cytometry analysis for TNNT2 from representative WT and TAB2 KO differentiation protocols prior to purification at day 13 (*n* = 3). The results are presented as means ± SEMs of 3 independent experiments. ns, not significant, unpaired 2-sided Student’s *t* test

In order to knock out the TAB2 gene as effectively as possible, we designed a knockout gRNA targeting the fourth exon of the TAB2 gene (Fig. 1A). Plasmid vectors containing gRNA and CRISPR/Cas9 were electroporated into human induced pluripotent stem cells. After amplification, DNA was extracted from the positive puromycin clones, and Sanger sequencing was carried out to confirm the knockout. Among the 14 clones analyzed, a single clone was identified, exhibiting a compound heterozygous knockout of TAB2. This was characterized by the insert of 1 and absence of 7 nucleotides on the two alleles, respectively (Fig. 1B). We reveal that the TAB2 knockout cell line retained embryonic stem cell morphology, such as high nucleo-plasmic ratios and densely packed clones (Fig. S1A). We further confirmed that TAB2^−/−^ cell lines expressed the human pluripotency-related genes (NANOG, OCT4, SOX-2 and REX1) as WT cells in RT-qPCR (Fig. S1B). Meanwhile, immunofluorescence staining revealed that the cell line exhibited the stemness markers OCT4 and SOX2 (Fig. 1C). Flow-cytometry analysis showed that there was no significant difference in SSEA4 positive rate between WT and KO cells (Fig. S1C, D). Moreover, we predicted the top three off-target sites for sgRNA using an online analysis software (crispr.mit.edu) and found no off-target mutations in TAB2^−/−^ hiPSCs by DNA sequencing (Additional file 2). Additionally, we confirmed through PCR that the CRISPR/Cas9 plasmid did not undergo random integration into the genome (Supplementary File 1).

### TAB2-deficient hiPSCs can differentiate into CMs

We first added small molecule compounds to TAB2-knocked out hiPSCs to enhance the induction of cardiomyocyte differentiation due to the crucial role of TAB2 in myocardial development (Burridge et al. 2014; Thienpont et al. 2010; Woods et al. 2022). Following this, the deletion of TAB2 protein in knockout (KO) cardiomyocytes was confirmed through Western Blot analysis at day 20, showing the absence of TAB2 protein expression (Fig. 1D). Subsequently, the differentiation efficiency of cardiomyocytes was assessed using flow cytometry. Notably, on the 13th day of myocardial differentiation, both wild-type (WT) and TAB2 KO-derived cardiomyocytes exhibited a differentiation efficiency of 83.5% (Fig. 1E, F). The purity of both WT and KO cardiomyocytes reached 98% by the 17th day after sugar starvation purification, with no significant discrepancies observed (Fig. S1E, F). These findings collectively indicate that the knockout of TAB2 does not impede the differentiation of cardiomyocytes.

### TAB2-deficient hiPSC-CMs replicate DCM characteristics in vitro

**Fig. 2:**
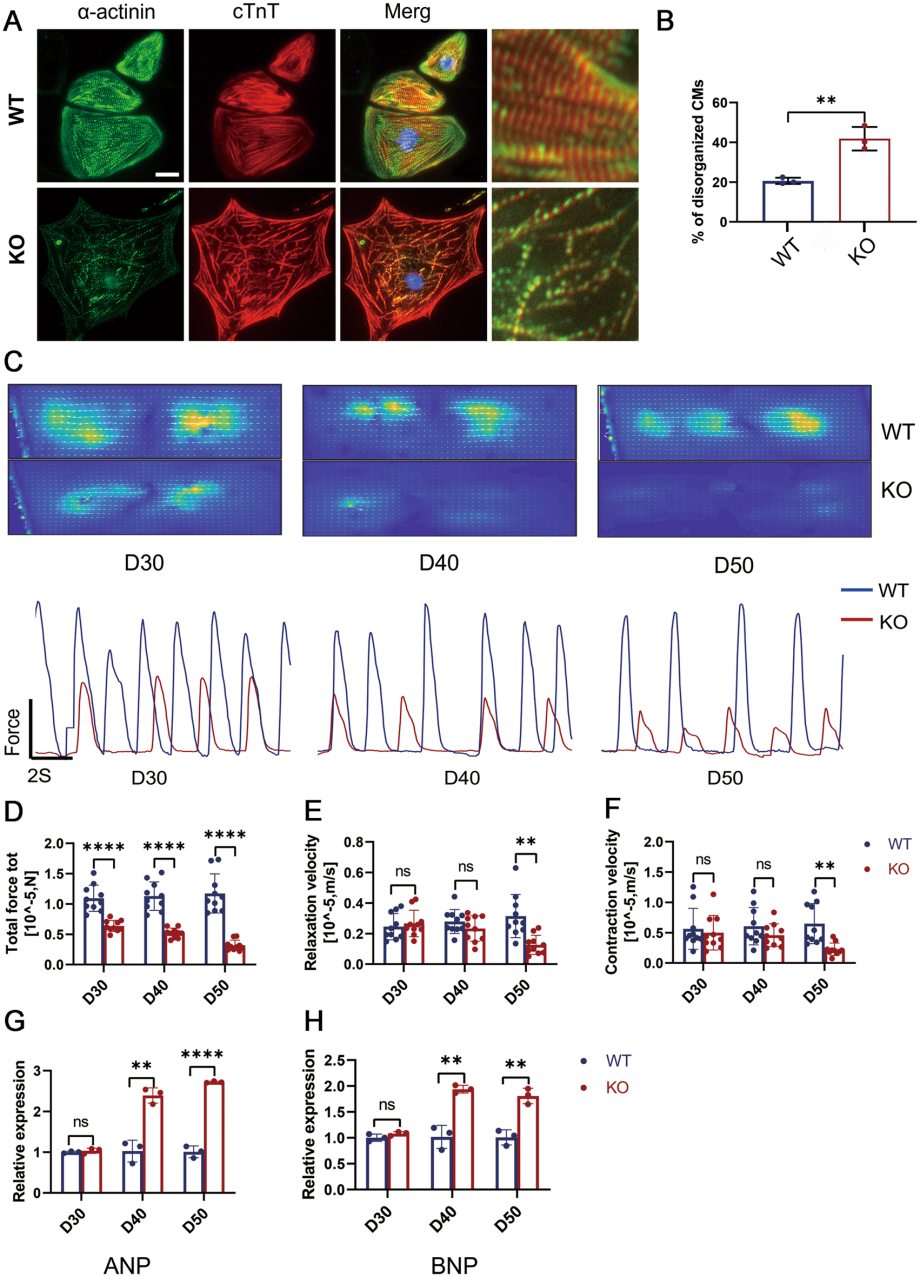
Phenotyping dilated cardiomyopathy in TAB2-deficient hiPSC-CMs. **A**,**B** Immunostaining of sarcomeric α-actinin (green) and cTnT (red) demonstrates sarcomeric disarray in WT (*n* = 269) and TAB2 KO (*n* = 285) CMs at day 30. Scale bar, 20 μm. **C** Representative heatmaps and line scan images in WT, TAB2-KO hiPSC-CMs myocardial contractility at days 30, 40, and 50. **D**–**F** Quantification of total force tot, relaxation velocity, and contraction velocity in WT and TAB2-KO hiPSC-CMs (*n* = 10 cells per group). **G**,**H** Quantitative real-time-PCR analysis of HF-related genes in WT, TAB2-KO hiPSC-CMs at days 30, 40, and 50 (*n* = 9). The results are presented as means ± SEMs of 3 independent experiments. * *P* < 0.05; ** *P* < 0.01; *** *P* < 0.001; ns, not significant, unpaired 2-sided Student’s *t* test

Following the findings by Eschenhagen and Carrier (2019), which identified myotome disturbance as a prevalent phenotype of Dilated Cardiomyopathy (DCM), our study focuses on the structural alterations in TAB2-knockout cardiomyocytes. Our investigation revealed that at the 30-day mark post-myocardial differentiation, TAB2-deficient cardiomyocytes exhibited a noticeable increase in the occurrence of sarcomere disorganization compared to wild-type (WT) cells, as evidenced by a significant rise in CTNT and actinin immunofluorescence staining (Fig. 2A, B).

Cardiomyocyte contraction plays a fundamental role in maintaining the heart’s essential function (Penefsky 1994), with one of the primary indicators of dilated cardiomyopathy (DCM) being the reduction in systolic function (Hinson et al. 2015). To assess the impact of TAB2 knockout on cardiomyocyte contractility, we conducted contractile force measurements on human induced pluripotent stem cell-derived cardiomyocytes (hiPSC-CMs) at 30, 40, and 50 days, and KO cells exhibit a phenotype of arrhythmia potential (Fig. 2C). The contractile amplitude of TAB2-KO cardiomyocytes exhibited a significant decrease compared to wild-type (WT) at the outset, and this decrease further amplified over time (Fig. 2D). Additionally, both the diastolic and contractile rates of TAB2-KO cardiomyocytes displayed noticeable declines as well (Fig. 2E, F). Furthermore, we also observe that other contractility parameters, such as mean contractility displacement, contractility time, and beating frequency, change with variations in culture time (Fig. S2A–H). From day 40 onwards, we observed a significant increase in the expression levels of ANP and BNP in TAB2 knockout cells, suggesting a progression towards heart failure (Fig. 2G, H).

### TAB2 deficiency leads to abnormal cardiac calcium transients

**Fig. 3:**
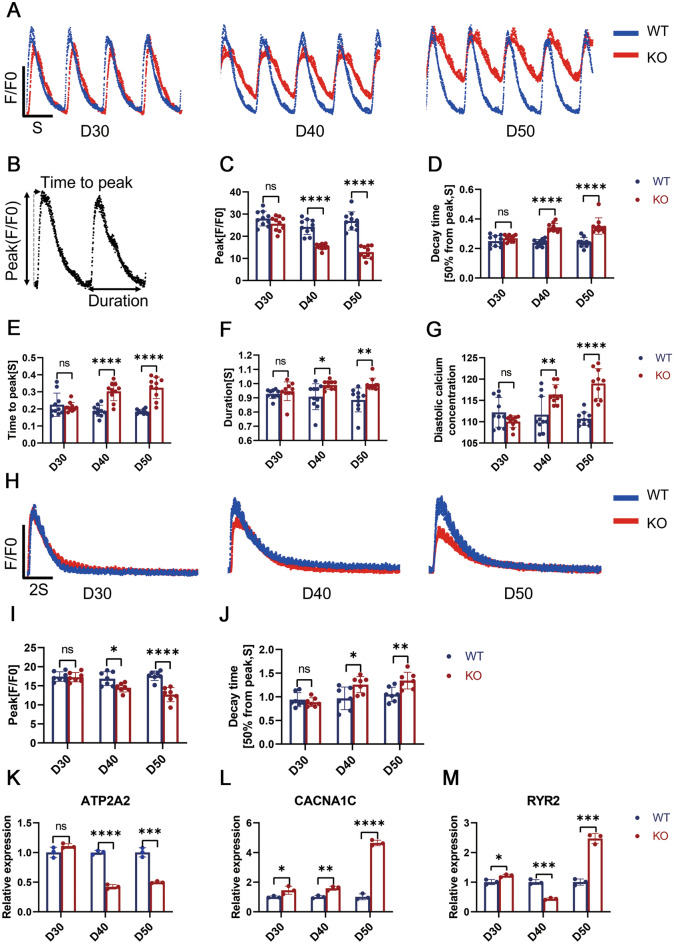
TAB2-deficient hiPSC-CMs exhibit abnormal Ca^2+^ handling properties. **A** Representative line-scan images in WT-GCaMP and TAB2 KO-GCaMP hiPSC-CMs at days 30, 40, and 50. **B** Space-averaged calcium transients showing parameters measured for analysis of calcium handling. **C**–**G** Quantification of peak, decay time, time to peak, calcium transient duration and calcium diastolic concentration in WT-GCaMP and TAB2 KO-GCaMP hiPSC-CMs (*n* = 10 cells per group). **H** Representative Ca^2+^ transients induced with 10 mM caffeine in Ca^2+^-free conditions between WT-GCaMP and TAB2 KO-GCaMP hiPSC-CMs at days 30, 40, and 50. **I**,**J** Peak amplitude and decay time in caffeine-evoked Ca^2+^ transients WT-GCaMP and TAB2 KO-GCaMP hiPSC-CMs (*n* = 7 cells per group). **K**–**M** Quantification of ATP2A2, CACNA1C and RYR2 normalized by GAPDH in WT and TAB2 KO hiPSC-CMs (*n* = 9). Results are presented as means ± S.E.M. of three independent experiments. * *P* < 0.05; ** *P* < 0.01; *** *P* < 0.001; **** *P* < 0.0001; ns, not significant, unpaired two-sided Student’s *t* test

Calcium transient abnormalities were often found in cardiomyocytes of dilated cardiomyopathy (Malkovskiy et al. 2020). Simultaneously, abnormal calcium processing is one of the primary causes of decreased contractility (Perreault et al. 1990). To investigate the impact of TAB2 knockout on the regulation of calcium in the heart, we integrated the calcium sensor Green fluorescent calmodulin 6 Rapid type (GCaMP6f) into the TAB2-KO cell line at the AAVS1 locus (Fig. S3A), aiming to detect any alterations in calcium transients at 30, 40 and 50 days (Fig. 3A). Initially, on day 30, there was no noticeable difference in calcium release amplitude (Fig. 3B) between wild-type (WT) and TAB2 knockout (KO) cardiomyocytes, which contradicted the observed change in contractility. However, by day 40, a decrease in calcium release amplitude was evident in KO hiPSC-CMs compared to the WT (Fig. 3C), accompanied by extended time to peak and decay time (Fig. 3D, E). Subsequently, by day 50, this trend became even more pronounced (Fig. 3C–E). In addition, we found that the calcium transient duration of the knockout cell line and the diastolic calcium ion concentration increased significantly on day 40 and continued to rise (Fig. 3F, G). These findings suggest that the abnormalities in calcium transients are likely not the primary cause of the reduced contractility observed in TAB2-knocked out hiPSC-CMs.

Since TAB2 knockouts lead to increased diastolic calcium concentrations, we conducted SR-caffeine-induced calcium release experiments on 30-, 40-, and 50-day-old wild-type (WT) and TAB2 KO-hiPSC CMs to further understand the properties of calcium handling (Fig. 3H). Consistent with the above calcium transient results, the calcium release amplitude and reuptake rate of KO cardiomyocytes began to decrease significantly at day 40 compared with WT (Fig. 3I, J). This indicates that TAB2 knockout in hiPSC-CMs reduced sarcoplasmic reticulum calcium storage and impaired membrane and SR Ca^2+^ pump function. Supporting this conclusion, there was increased gene expression of SERCA2a, CACNA1C and RYR2 (Fig. 3K–M). This provides further evidence of the impaired calcium handling in the TAB2 KO cells. However, it was confirmed once again that calcium ion homeostasis imbalance was not the priming cause of the development of dilated cardiomyopathy (DCM) resulting from TAB2 deficiency.

### TAB2 deficiency leads to mitochondrial damage reduced energy metabolism

**Fig. 4:**
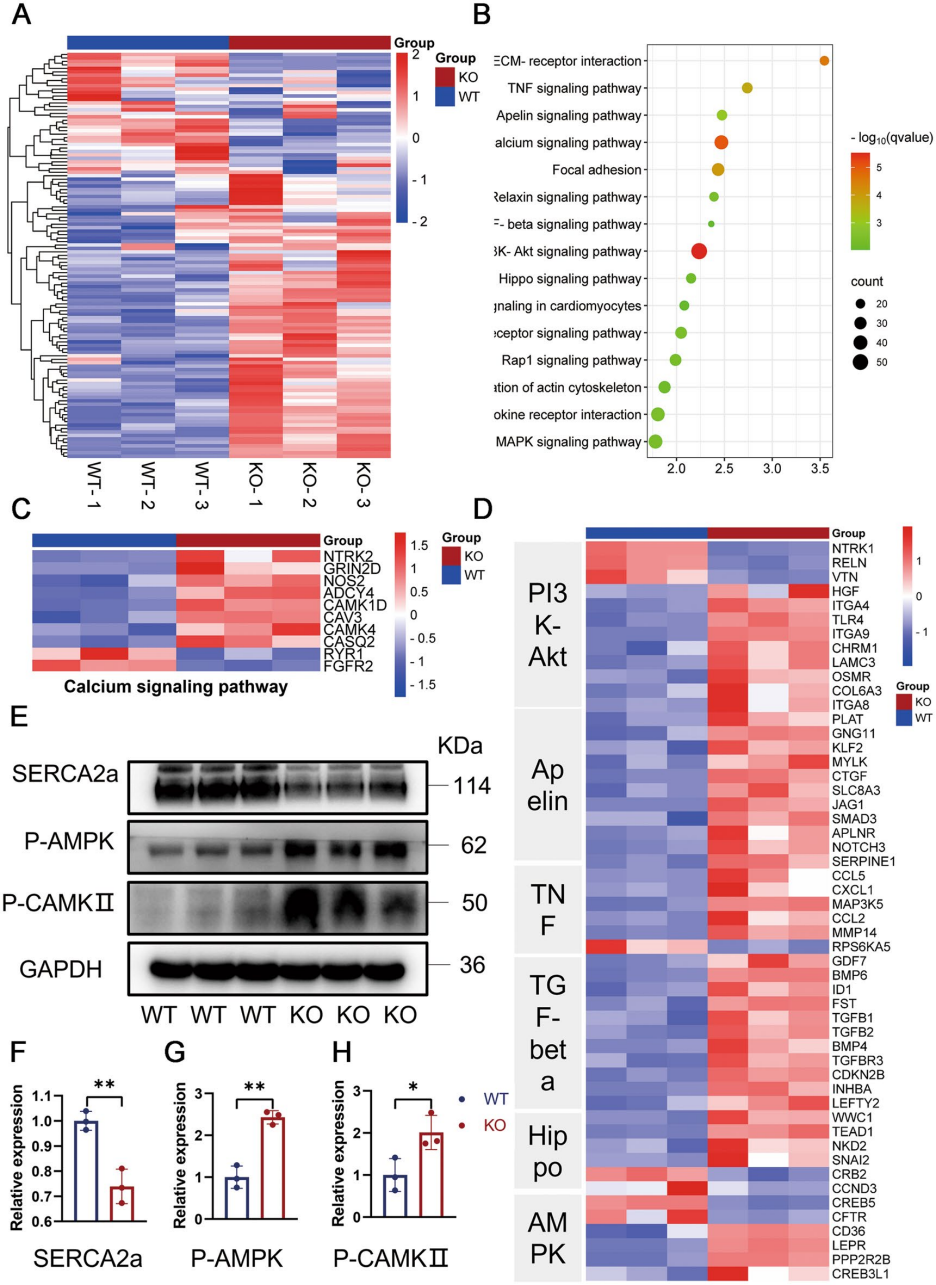
Transcriptome analysis in TAB2 deficient and WT CMs. **A** RNA-Seq was used to detect differentially expressed genes in WT and KO hiPSC-CMs (*n* = 3). **B** KEGG analysis of pathway enrichment changes in KO hiPSC-CMs. **C** Heatmap shows expression changes of genes related to calcium signaling pathway in WT and KO CMs at days 40. **D** Heatmap shows expression changes of genes related to PI3K-Akt, Apelin TNF, TGF-beta, Hippo and AMPK signaling pathway in WT and KO CMs at days 40. **E**–**H** Western blot shows expression of SERCA2a, P-AMPK and P-CAMKII proteins in WT and KO CMs, respectively (*n* = 3). Results are presented as means ± S.E.M. of three independent experiments. * *P* < 0.05; ** *P* < 0.01; unpaired two-sided Student’s *t* test

To gain insight into the molecular mechanism via which TAB2 wiped out the phenotype associated with dilated cardiomyopathy in cardiomyocytes, we employed RNA-Seq techniques to detect and analyze variations in gene expression profiles between groups of WT and KO cardiomyocytes (Fig. 4A). The results revealed significant differences between WT and KO cardiomyocytes (Fig. S3B), with 1381 genes altered in KO cells compared to WT, including 983 genes with significantly increased expression and 398 genes with dramatically decreased expression (Fig. S3C). Then, we conducted a Gene Ontology (GO) analysis on the sequencing results and found that the cardiomyocytes with TAB2 knockout exhibited significant pathological growth and remodeling (Fig. S3D). Subsequently, we performed KEGG (Kyoto encyclopedia of genes and genomes) analysis on the sequencing results gathered from WT and KO cells to explore the differences in the activity state of signaling pathways between the two groups (Fig. 4B). Cardiomyopathy and heart failure-related pathways were significantly enriched in KO cardiomyocytes compared to WT cardiomyocytes. Furthermore, KO cardiomyocytes were considerably enriched in calcium signal-related pathways, which was in line with the results of our previous calcium transient study (Fig. 4C). Perhaps more importantly, KO cardiomyocytes exhibited significantly elevated levels of mitochondria-associated pathways, such as PI3K-AKT, Apelin and TNF signaling pathways (Fig. 4D), as well as apoptosis and necroptosis (Fig. S3E, F), suggesting mitochondrial damage in TAB2-deficient cardiomyocytes. In addition, we also confirmed that the expression levels of signaling pathway-related proteins SERCA2a, P-AMPK and P-CAMKII altered significantly in knockout cell lines compared to wild type (Fig. 4E–H).

**Fig. 5:**
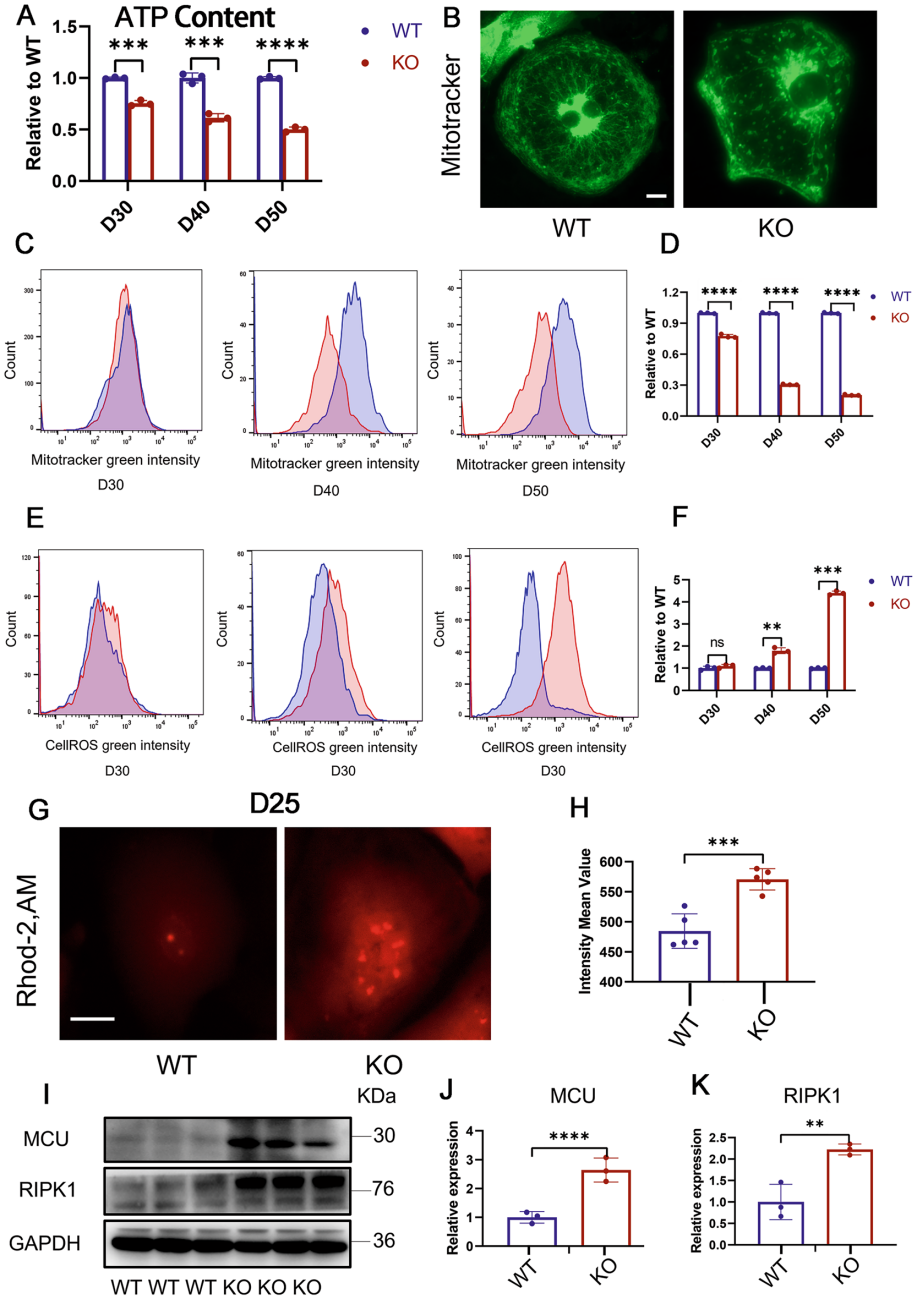
Mitochondrial damage and calcium overload in TAB2-deficient CMs. **A** ATP content measure in WT and KO cardiomyocytes at day 30, 40 and 50 (*n* = 3). **B** Immunostaining of Mitotracker Green in WT and KO hiPSC-CMs at day 30. **C**,**D** Quantification of Mitotracker Green intensity obtained by flow cytometry in TAB2-KO hiPSC-CMs at days 30, 40, and 50 as compared with WT hiPSC-CMs (*n* = 3). **E**,**F** Quantification of Cell ROS Green intensity obtained by flow cytometry in TAB2-KO hiPSC-CMs at days 30, 40 and 50 as compared with WT hiPSC-CMs (*n* = 3). **G** Immunostaining of Rhod-2, AM in WT and KO hiPSC-CMs at day 25. **H** Quantitative analysis of Rhod-2 AM immunofluorescence intensity (*n* = 13 cells per group). **I**–**K** Immunoblot analysis of RIPK1 and MCU in WT and TAB2 KO hiPSC-CMs (*n* = 3). Results are presented as means ± S.E.M. of three independent experiments. * *P* < 0.05; ** *P* < 0.01; *** *P* < 0.001; **** *P* < 0.0001; ns, not significant, unpaired two-sided Student’s *t* test

The heart primarily depends on mitochondrial oxidative metabolism to fulfill approximately 95% of its energy requirements (Doenst et al. 2013). Therefore, insufficient cellular energy is also a significant reason for the notable reduction in contractile force. ATP, as a direct source of energy, is our primary detection index. We measured ATP levels in both groups of cells at day 30, 40 and 50 (Fig. 5A). These results indicate that TAB2 knockout cardiomyocytes have a reduced energy supply, which manifests as a heart failure phenotype.

To accurately assess the mitochondrial density of cardiomyocytes, the 30-day-old cells were stained using immunofluorescence. The mitochondrial content of KO iPSC-CMs was significantly reduced compared to the control group (Fig. 5B). At the same time, the fluorescence intensity of WT and KO cardiomyocytes incubated with MitoTracker was detected using flow cytometry (Fig. 5C, D). We observed a significant decrease in the fluorescence intensity of mitochondria in the experimental group at 30 days, which continued to deteriorate over time. Next, we also measured the mitochondrial content of WT and KO iPSC-CMs by comparing the ratio of mitochondria-specific ND1 and ND2 genes to the housekeeping gene ACTB at 30, 40, and 50 days in the two sets of cells (Fig. S4A, B). Similar to flow cytometry, the mitochondrial DNA (mtDNA) to nuclear DNA (nDNA) ratio of KO cardiomyocytes was significantly lower than that of WT cells at 30 days.

### TAB2 deficiency causes mitochondrial calcium overload through MCU activation

Mitochondria play a crucial role as the primary energy source of cells while also regulating intracellular Ca^2+^ homeostasis, intermediate metabolite/ROS production, and various cellular processes (Wang et al. 2018). ROS, generated by cardiomyocytes primarily from mitochondria during aerobic metabolism (Chouchani et al. 2014; Scialo et al. 2016), have been linked to several heart conditions. To assess ROS levels, we utilized DCFH-DA to measure ROS content in WT and KO cardiomyocytes (Fig. 5E, F). Notably, at day 30, there was no significant difference observed between WT and KO cells. However, at day 40, an evident increase in fluorescence intensity was noted in KO cells, suggesting that ROS may not be an initial factor in mitochondrial damage progression. In addition, the cells were immunofluorescently stained with DCFH-DA probes on day 40 to re-validate the flow cytometry results and confirm the increased ROS levels in the KO cells (Fig. S4C, D).

The pivotal transduction signals between the sarcoplasmic reticulum (SR) and mitochondria are Ca^2+^ and ROS (Li et al. 2015; Yan et al. 2008), with mitochondrial calcium levels governing various mitochondrial functions. Utilizing RHOD-2, AM fluorescence probes, we evaluated mitochondrial calcium ion levels in WT and KO cardiomyocytes on day 25. Immunofluorescence imaging revealed a considerable rise in mitochondrial calcium content in KO cells (Fig. 5G, H). Flow cytometry measurement of RHOD fluorescence intensity further confirmed the significant increase in KO cells on day 25 (Fig. S4E, F). These observations indicate that mitochondrial calcium overload in TAB2 gene knockout cardiomyocytes preceded the ROS increase.

Mitochondrial Ca^2+^ influx primarily occurs through the mitochondrial calcium uniporter (MCU), while the mitochondrial Na^+^–Ca^2+^–Li^+^ exchanger (mNCLX) facilitates calcium expulsion (Brown and O’Rourke 2010; Campanella et al. 2004; Palty et al. 2010). Notably, we observed a pronounced elevation in MCU protein content in the mitochondrial inner membrane of KO cardiomyocytes, indicating increased MCU expression (Fig. 5I, J). Previous studies have shown that RIPK1 enhances MCU expression by interacting with it (Zeng et al. 2018), and in TAB2 knockout mice, TAB2 deficiency promotes RIPK1-dependent apoptosis and necrotic apoptosis by upregulating RIPK1 expression and activation (Yin et al. 2022). Consistently, we found elevated RIPK1 expression in KO cells during our experiments (Fig. 5I, K).

To further confirm that the knockout of TAB2 results in impaired cardiomyocyte function independent of cell differentiation, we administered small interfering RNA to reduce TAB2 expression (Fig. S5A) on day 20 following cardiomyocyte differentiation. We assessed changes in contractility (Fig. S5B–E), calcium transients (Fig. S5F–I), mitochondrial content (Fig. S5J, K), and ROS levels (Fig. S5L, M) at day 40. Our findings indicate that the knockdown of TAB2 expression following cell differentiation still resulted in a phenotype characterized by impaired cardiac function.

### RIPK1 inhibitors prevent TAB2-knocked out cardiomyocytes from developing DCM phenotypes

**Fig. 6:**
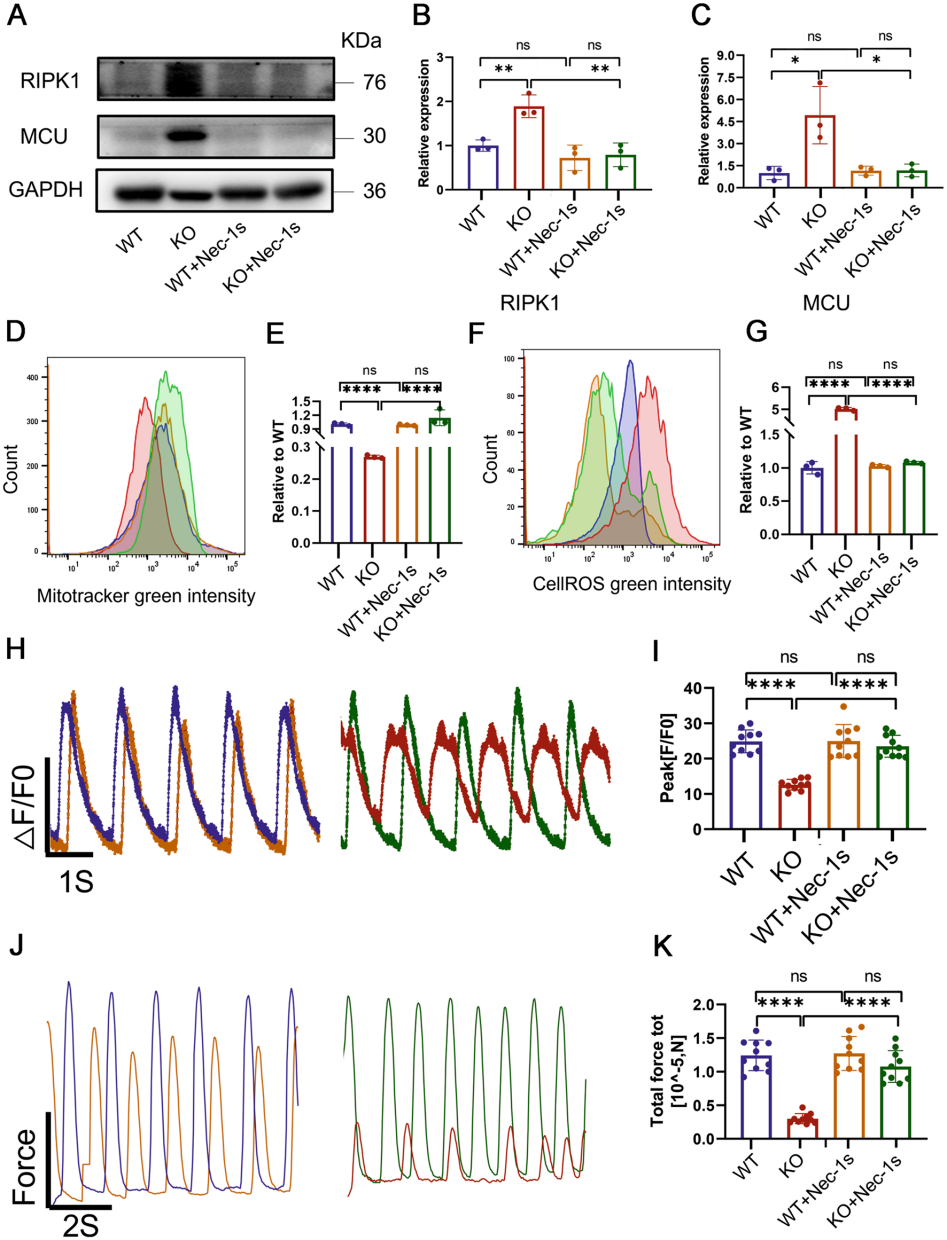
Nec-1s rescued DCM phenotype in TAB2-deficient CMs. **A**–**C** Immunoblot analysis of RIPK1 and MCU in WT, TAB2-KO, WT + Nec-1s, and TAB2-KO + Nec-1s hiPSC-CMs at day 50 (*n* = 3). **D**,**E** Quantification of MitoTracker Green intensity obtained by flow cytometry fluorescence intensity in WT, TAB2-KO, WT + Nec-1s, and TAB2-KO + Nec-1s hiPSC CMs at day 50 (*n* = 3). **F**,**G** Quantification of Cell ROS (green) intensity obtained by flow cytometry fluorescence intensity in WT, TAB2-KO, WT + Nec-1s, and TAB2-KO + Nec-1s hiPSC CMs at day 50 (*n* = 3). **H** Representative line scan images in WT-GCaMP, TAB2 KO-GCaMP, WT-GCaMP + Nec-1s, and TAB2-KO-GCaMP + Nec-1s hiPSC-CMs at day 50. **I** Quantification of peak in WT-GCaMP, TAB2 KO-GCaMP, WT-GCaMP + Nec-1s, and TAB2-KO-GCaMP + Nec-1s hiPSC-CMs at day 50 (*n* = 10 cells per group). **J** Representative line scan images in WT, TAB2-KO, WT + Nec-1s, and TAB2-KO + Nec-1s hiPSC CMs myocardial contractility at day 50. **K** Quantification of total force tot in WT, TAB2-KO, WT + Nec-1s, and TAB2-KO + Nec-1s hiPSC CMs at day 50 (*n* = 10 cells per group). The results are presented as means ± SEMs of 3 independent experiments. * *P* < 0.05; ** *P* < 0.01; *** *P* < 0.001; ns, not significant, unpaired 2-sided Student’s *t* test. One-way ANOVA and least significant difference (LSD) test were used to compare the parameters between groups

Considering that mitochondrial calcium overload triggered by RIPK1 activation precedes the DCM-associated phenotype, we explored whether RIPK1 inhibitors could potentially halt the progression of DCM and HF phenotypes in TAB2-knockout cardiomyocytes. Nec-1, an ATP-competitive allosteric inhibitor of RIPK1, is also known to significantly inhibit indoleamine 2,3-dioxygenase (IDO), which breaks down the essential amino acid tryptophan into kynurenine. The IDO-kynurenine pathway plays a critical role in regulating both the innate and adaptive immune systems and is also involved in neuroprotection (Liu et al. 2009). Nec-1s, also known as 7-Cl-O-Nec-1, is an optimized, more stable version of Nec-1 and was the inhibitor investigated in this study. Unlike Nec-1, Nec-1s specifically inhibits RIPK1 without affecting IDO activity (Yu et al. 2021). Considering that the cell phenotype is most stable on day 50, which aligns with clinical treatment practices, we selected day 50 for drug intervention. Cardiomyocytes were treated with a 10 μmol concentration of Nec-1s for 48 h on day 50 for both WT and KO groups. Post-intervention, we observed a decrease in the expression levels of RIPK1 and MCU in KO cardiomyocytes, with no significant difference compared to the WT group (Fig. 6A–C). Flow cytometry evaluation of mitochondrial content and ROS levels indicated that Nec-1s administration reduced mitochondrial damage and ROS production in TAB2-deficient hiPSC-CMs (Fig. 6D–G). At the same time, to further confirm that Nec-1s intervention can significantly improve the phenotype of TAB2-deficient cardiomyocytes, we performed qPCR experiments to detect the expression changes of mitochondrial genes ND1 and ND2. The results showed that after the intervention, the expression of ND1 and ND2 significantly increased (Fig. S6A, B). Additionally, Nec-1s intervention restored the calcium processing capacity of KO cardiomyocytes, manifesting in increased amplitudes of calcium (Fig. 6H, I), as well as an increase in the rate of calcium release and recovery, a decrease in calcium ion concentration during diastole (Fig. S6C–E). Furthermore, contractile force changes were detected in KO cardiomyocytes after the drug intervention (Fig. 6J, K), showing an increase in contractile and diastolic speed in KO cells treated with Nec-1s (Fig. S6F, G). Other parameters of contractile force were also significantly improved (Fig. S6H–N). These findings once again confirm that intervention with Nec-1s could potentially rescue TAB2 knockouts from DCM and HF phenotypes.

## Discussion

**Fig. 7:**
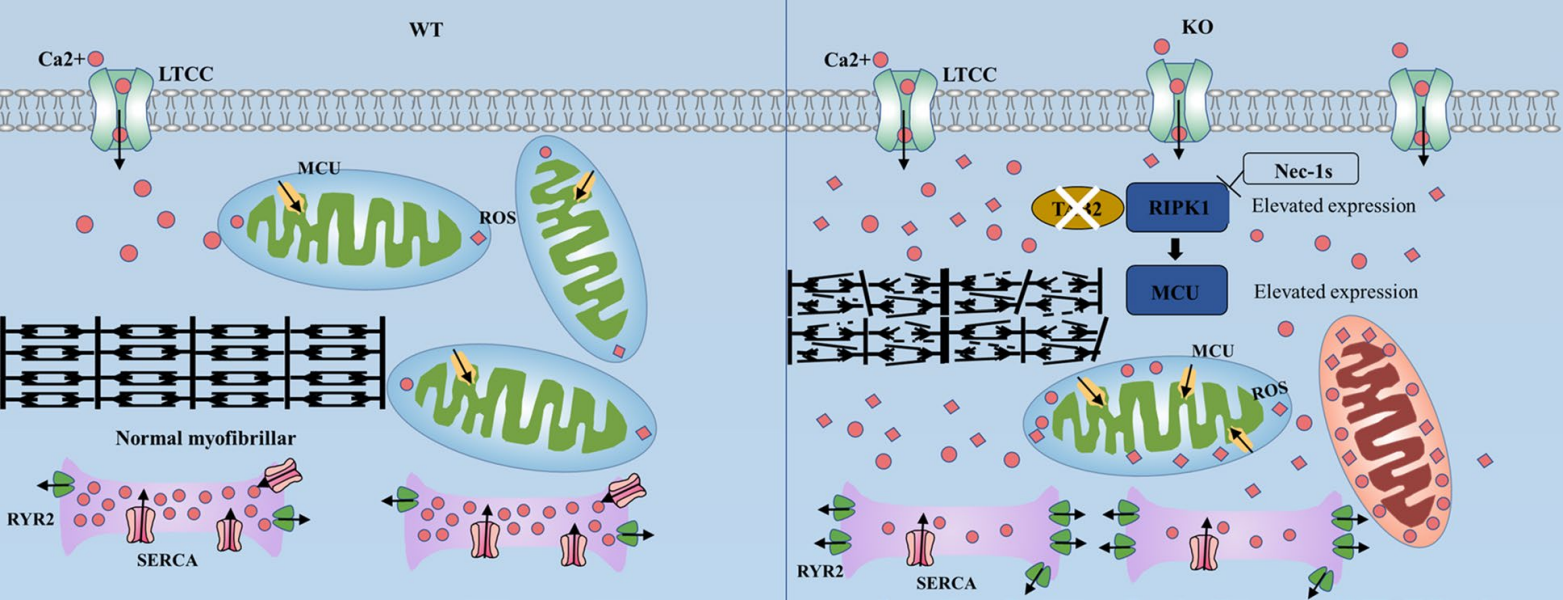
A model for mechanisms of cardiac dilated in TAB2 deficient cardiomyocytes. TAB2 deficiency leads to the activation and increased expression of RIPK1 and MCU proteins, resulting in mitochondrial calcium overload. RIPK1, receptor-interacting protein kinase 1; MCU, mitochondrial calcium uniporter

In this study, we used CRISPR/Cas9 gene-editing technology to create TAB2 compound heterozygous knockout induced pluripotent stem cell lines. Subsequently, we induced their differentiation into cardiomyocytes to establish an in vitro TAB2-knockout cardiomyopathy model. TAB2 knocked out cardiomyocytes showed DCM and HF characteristics such as reduced contractility, myotome disturbance, and abnormal calcium processing in vitro. This model helped us find a new pathogenic mechanism of DCM. We found that mitochondrial calcium overload, caused by increased expression of RIPK1 and MCU, is the core mechanism of DCM induced by TAB2 knockout. Early application of the RIPK1 inhibitor Nec-1s can block the development of DCM (Fig. 7).

We discovered that mitochondrial damage happens first, which is believed to be the primary mechanism. The main causes of mitochondrial damage were an increase in ROS and calcium excess in mitochondria. However, our findings revealed that mitochondrial calcium content increased dramatically at day 30 of cardiomyocyte development, whereas ROS did not alter much, indicating mitochondrial calcium overload as the initiating factor. Mitochondrial calcium overload is mostly caused by increased MCU expression in the mitochondrial intima or cytoplasmic calcium levels. We observed the calcium transient and discovered that the cytoplasmic calcium concentration had not increased by day 30. Furthermore, it has been determined that TAB2 deficiency results in increased expression of RIPK1, which interacts with MCU. RIPK1 serves as a pivotal regulator of cellular death and inflammatory processes (Van Eeckhoutte et al. 2023). It has the capacity to influence the mechanisms of cell death through its interactions with autophagy-related proteins, including RUBCNL/PACER (Rojas-Rivera et al. 2024). Additionally, RIPK1 can engage with proteins such as Fas-associating protein with a novel death domain (FADD) and caspase-8, thereby facilitating the induction of both apoptosis and necroptosis. The functionality of RIPK1 is contingent upon post-translational modifications and its specific cellular localization. The preservation of a dynamic equilibrium between autophagy and apoptosis is essential for cellular health and the organism’s response to pathological states (Bell and Walsh 2009). Based on our study, we suggest that the initial excitation event is caused by mitochondrial calcium overload resulting from the increased expression of RIPK1 and MCU. Mitochondria serve as a scaffold integrating pro-apoptotic and anti-apoptotic signals (Bravo-Sagua et al. 2013; Czabotar et al. 2014). When mitochondrial calcium overload makes them sensitive to pro-apoptotic stimulation, it reduces the threshold of mitochondrial membrane permeability transition pore opening, leading to mitochondrial collapse (Bravo-Sagua et al. 2017). The electron transport chain is the primary source of ROS (Turrens 2003), and the elevation of mitochondrial calcium ions triggers the generation of ROS (Fearon 2006). At day 40, the level of total ROS increased due to the rise in calcium ions in mitochondria. The increased ROS interacts with calcium ions to promote additional mitochondrial damage. Therefore, compared with WT, ROS levels in KO cells at 50 days were significantly increased, while the fluorescence intensity of MitoTracker and the ratio of mitochondrial DNA to genomic DNA was significantly decreased. At the same time, mitochondria are the core of cellular energy metabolism (Wang et al. 2018) and can produce energy through oxidative phosphorylation.95% of ATP consumed by the heart comes from mitochondria (Kolwicz et al. 2013). In TAB2-knockout cardiomyocytes, mitochondrial damage leads to reduced ATP synthesis and increased P-AMPK protein content.

Mitochondria are the centers of energy metabolism and are key to regulating the normal contraction of the heart. After early mitochondrial damage, the energy supply of KO cardiomyocytes was insufficient, leading to a significant decrease in contractile force at 30 days. There was no change in systolic and diastolic speed, and no significant difference was observed in cellular calcium handling. This may be because myofilament sliding is more affected by energy metabolism than sarcoplasmic reticulum calcium reuptake. In cardiomyocytes, calcium ions are the core elements of excitation–contraction coupling and also participate in a variety of intracellular signaling cascades (Dewenter et al. 2017), acting as central regulators of cardiomyocyte contraction. At day 40, the continuous reduction of mitochondrial ATP production led to the blockage of Ca^2+^ reuptake by the sarcoplasmic reticulum through SERCA2a. This resulted in an increase in cytoplasmic calcium ion concentration and decrease in sarcoplasmic reticulum calcium reserves. We know that an increase in cytoplasmic calcium ion concentration can activate calcineurin (Creamer 2020); However, it was previously reported that TAB2, as an anchor protein, connects TAK1 with regulator of calcineurin 1 (RCAN1), facilitates the phosphorylation of RCAN1 by TAK1 at serine 94 and 136, and promotes the expression of calcineurin-NFAT signaling pathway (Liu et al. 2009). Therefore, we should have assessed calcineurin activity here, comparing the effects of TAB2 loss and elevated cytoplasmic calcium ion levels on its activation. The reduction in the amplitude of calcium transient release, prolonged decay time, and abnormal calcium processing further decreased the contractility of KO cardiomyocytes. Moreover, the continuous reduction of calcium recovery led to a significant decrease in the contraction velocity and diastolic velocity of TAB2^−/−^ hiPSCs, along with a decrease in the peak contractile force on day 50.

Since mitochondrial calcium overload caused by increased activation and expression of RIPK1 is the core mechanism of DCM induced by TAB2 knockdown in cardiomyocytes, we hypothesized that the administration of the RIPK1 inhibitor Nec-1s to KO cardiomyocytes would ameliorate the DCM phenotype. In this experiment, when DCM was about to develop into HF, Nec-1s was applied 48 h in advance. We found that KO cardiomyocytes after intervention, significantly reduced the expression of RIPK1 and MCU, largely blocked mitochondrial damage and the production of reactive oxygen species, and significantly improved the contractility and calcium processing of KO cells.

## Conclusion

In conclusion, we successfully established a human model of TAB2 knockout in vitro. In early TAB2 knockout cells, RIPK1 is activated, leading to increased expression. The interaction with MCU promotes the enhancement of MCU expression, resulting in mitochondrial calcium overload. Excessive calcium accumulation in mitochondria leads to increased ROS production, early mitochondrial damage, and decreased ATP content. These factors continue to interact, causing more serious mitochondrial damage. It even affects cell survival. Simultaneously, mitochondrial damage caused by an energy supply shortage leads to a decrease in early contractile force. Additionally, the middle sarcoplasmic reticulum experiences abnormal calcium reuptake, resulting in reduced calcium transient amplitude and prolonged calcium recovery time. This, in turn, affects the contractile force of cells in the middle and late stages, leading to further decreases in contraction and diastole speed. However, there are still major limitations in our study. First, the cardiomyocytes derived from induced pluripotent stem cells cultured in our lab are relatively naive compared to those derived from other sources. Second, our cultured cardiomyocytes could not accurately replicate the occurrence and development of DCM in tissues and organs in vitro. Third, our experiment can only demonstrate that TAB2 knockout can result in DCM. However, in clinical practice, the gene mutation of TAB2 is currently observed as a simple heterozygous mutation. Whether DCM caused by a TAB2 mutation in clinical practice is due to this mechanism, we are not certain. Despite some limitations, we have, for the first time, established TAB2-knockout iPSC-derived cardiomyocytes using CRISPR/Cas9, discovered a novel mechanism of TAB2-knockout DCM, and improved the phenotype by inhibiting RIPK1. It introduces a new approach for treating DCM.

## Supplementary Information

Additional file 1.

Additional file 2.

Additional file 3.

## Data Availability

The datasets used and/or analyzed during the current study are available from the corresponding author on reasonable request.

## Abbreviations

ACTB: Actin beta
AMPK: AMP-activated protein kinase
ANP: Atrial natriuretic peptide
ATP2A2: ATPase sarcoplasmic reticulum Ca^2+^ transporting 2
BNP: B-type natriuretic peptide
CACNA1C: Calcium voltage-gated channel subunit alpha1 C
CDS: Coding sequence
CHD: Congenital heart disease
CRISPR/Cas9: Clustered regularly interspaced short palindromic repeats
DCM: Dilated cardiomyopathy
GCaMP6f: The green fluorescent calcium-modulated protein 6 fast type
gRNA: Guide RNA
HCM: Hypertrophic cardiomyopathy
HF: Heart failure
hiPSC-CM: Human induced pluripotent stem cell derived cardiomyocytes
iPSC: Induced pluripotent stem cell
KO: Knockout
MCU: Mitochondrial calcium uniporter
RIPK1: Receptor-interacting protein kinase 1
SERCA: Sarco/endoplasmic reticulum Ca^2+^-ATPase
ROS: Reactive oxygen species
RYR2: Ryanodine receptor 2
TAB2: TGF-β-activated kinase 1 binding protein 2
TAK1: TGF-β-activated kinase 1
TNNT2: Troponin T2
WT: Wild-type
