# Supplementary material for: TAB2 deficiency induces dilated cardiomyopathy by promoting mitochondrial calcium overload in human iPSC-derived cardiomyocytes
Source: Mol Med. 2025 Feb 4;31:42. doi: 10.1186/s10020-025-01103-x (PMC11792723; doi:10.1186/s10020-025-01103-x)
Supplement: Supplementary file 1 — Additional file 1. [file 10020_2025_1103_MOESM1_ESM.docx]

**Supplement Fig 1** The pluripotency of TAB2 knockout iPSCs.


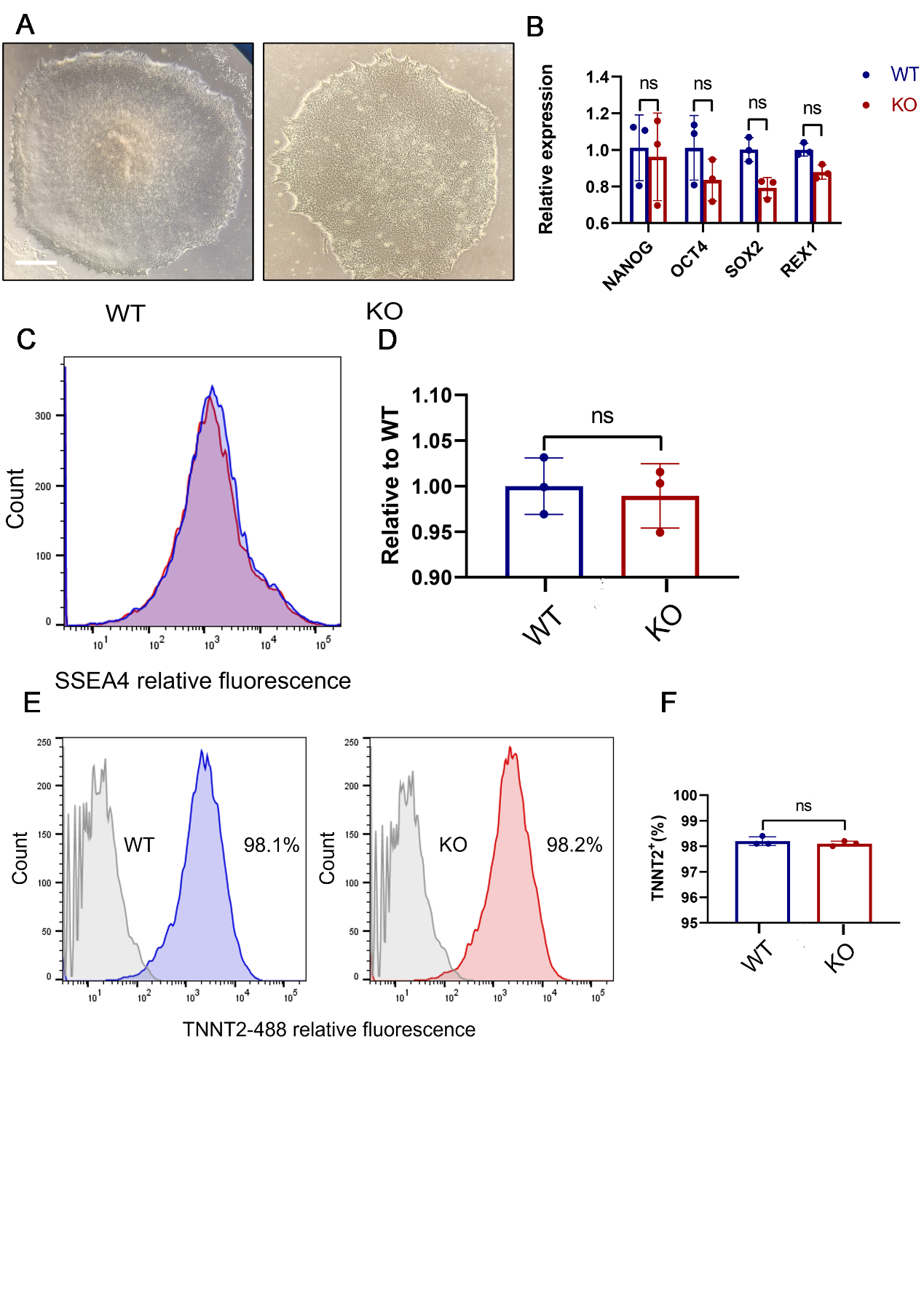


**Supplement Fig 2** Other contractility data of TAB2 knockout iPSC-CMs.


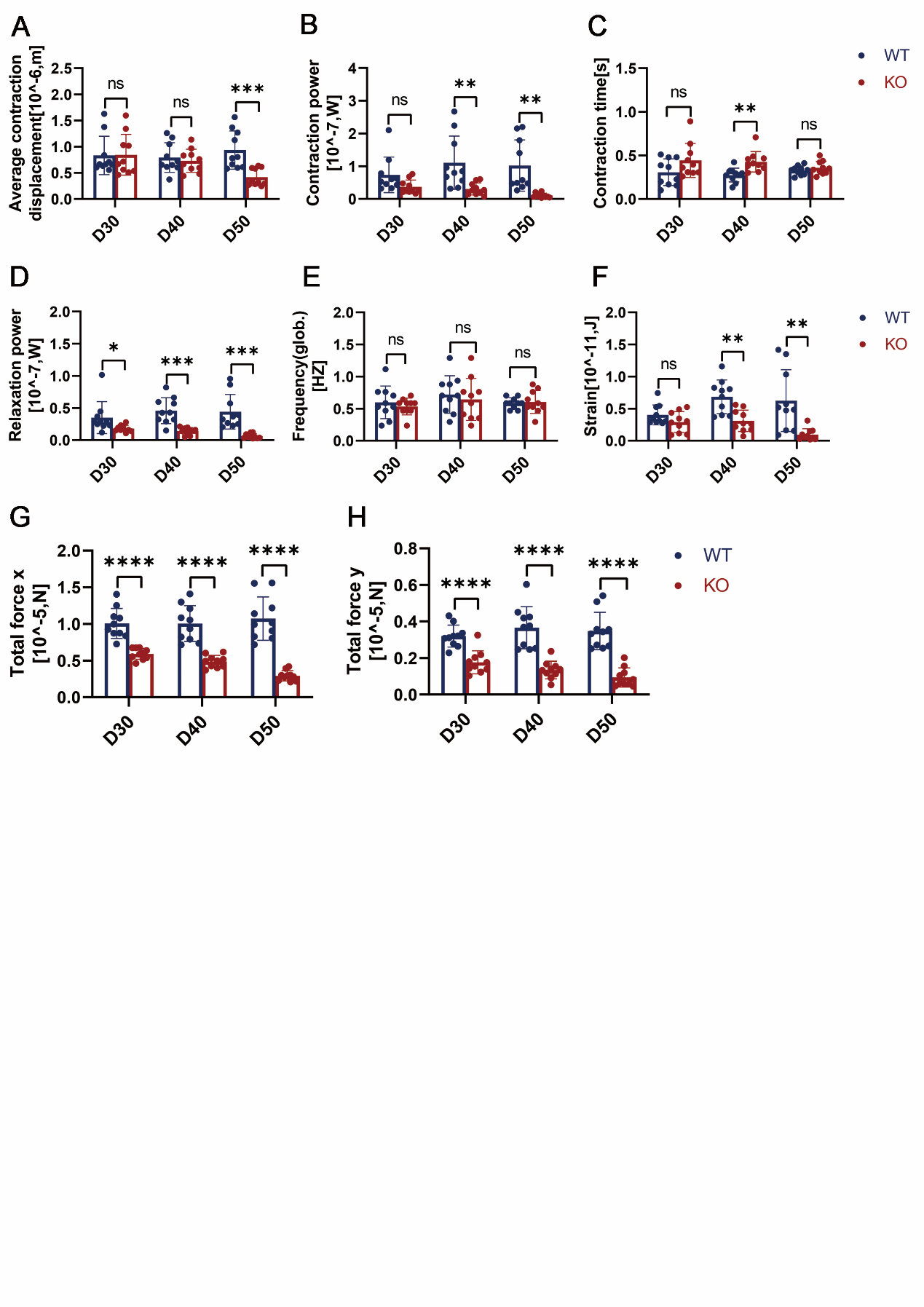


**Supplement Fig 3** Comparison of transcriptome sequencing between WT and TAB2 ko cell lines.


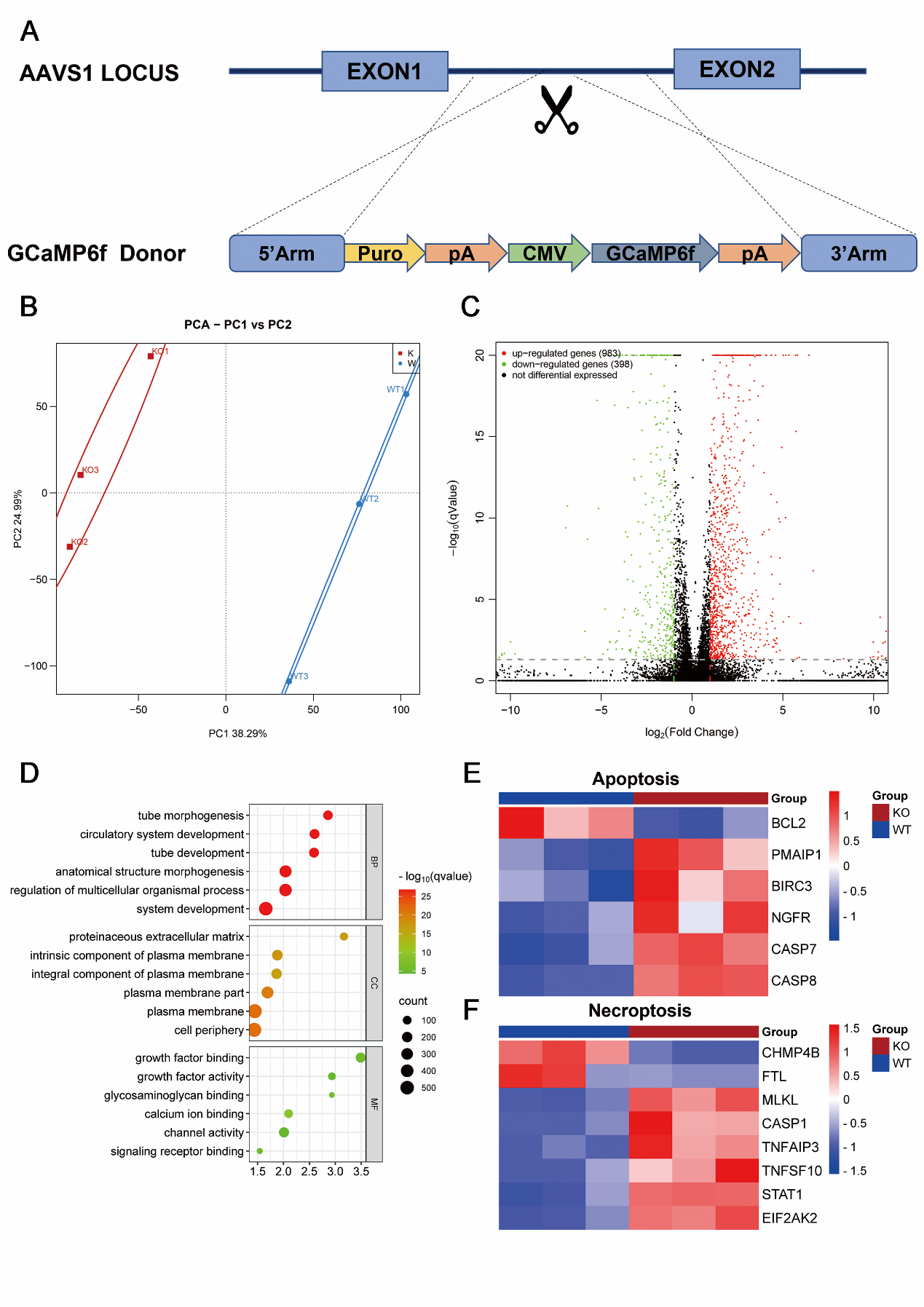


**Supplement Fig 4** Other phenotypic changes of mitochondria damage in TAB2 deficient cell lines.


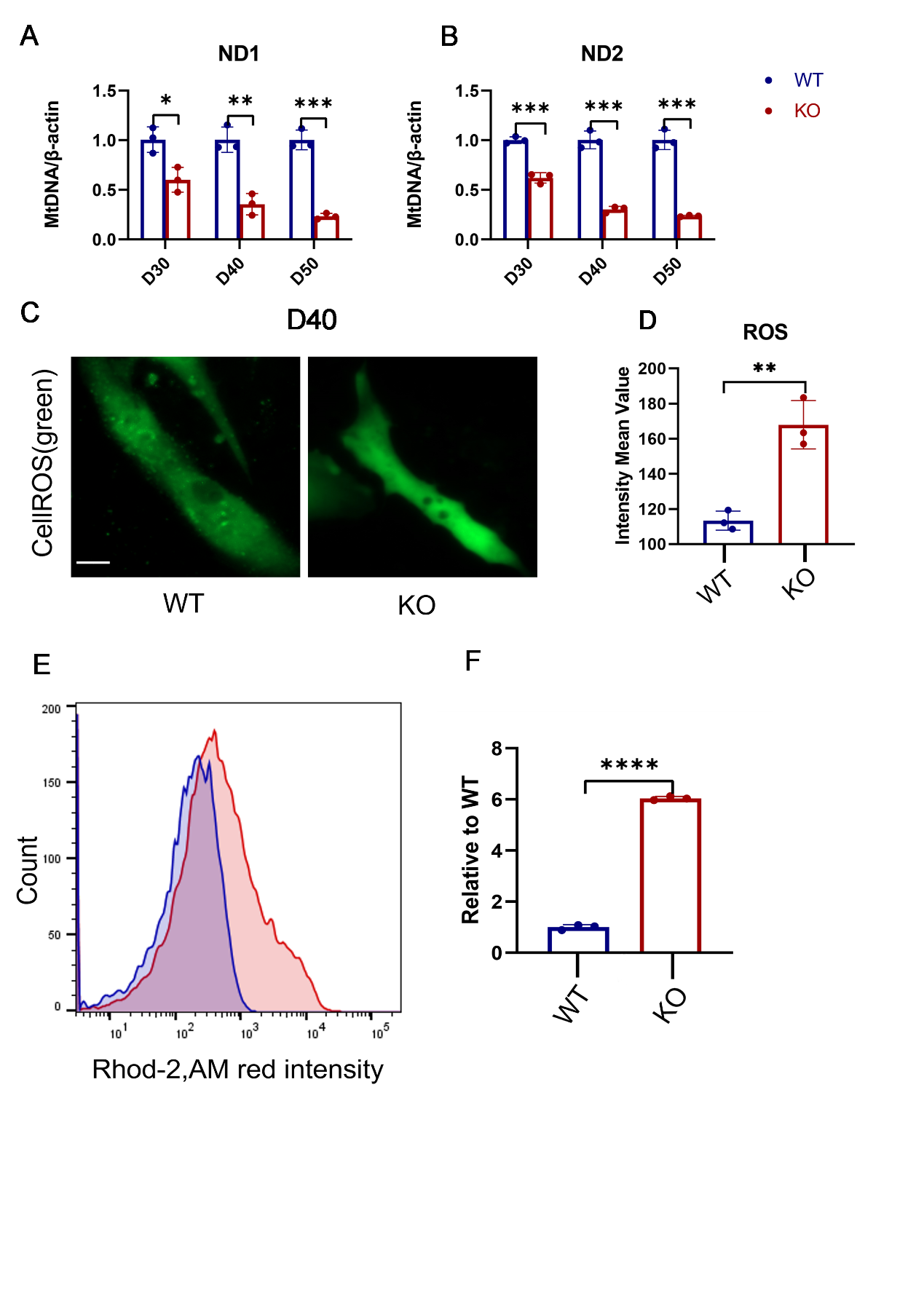


**Supplement Fig 5** The interference of the TAB2 gene in cardiomyocytes leads to impaired cardiac function.


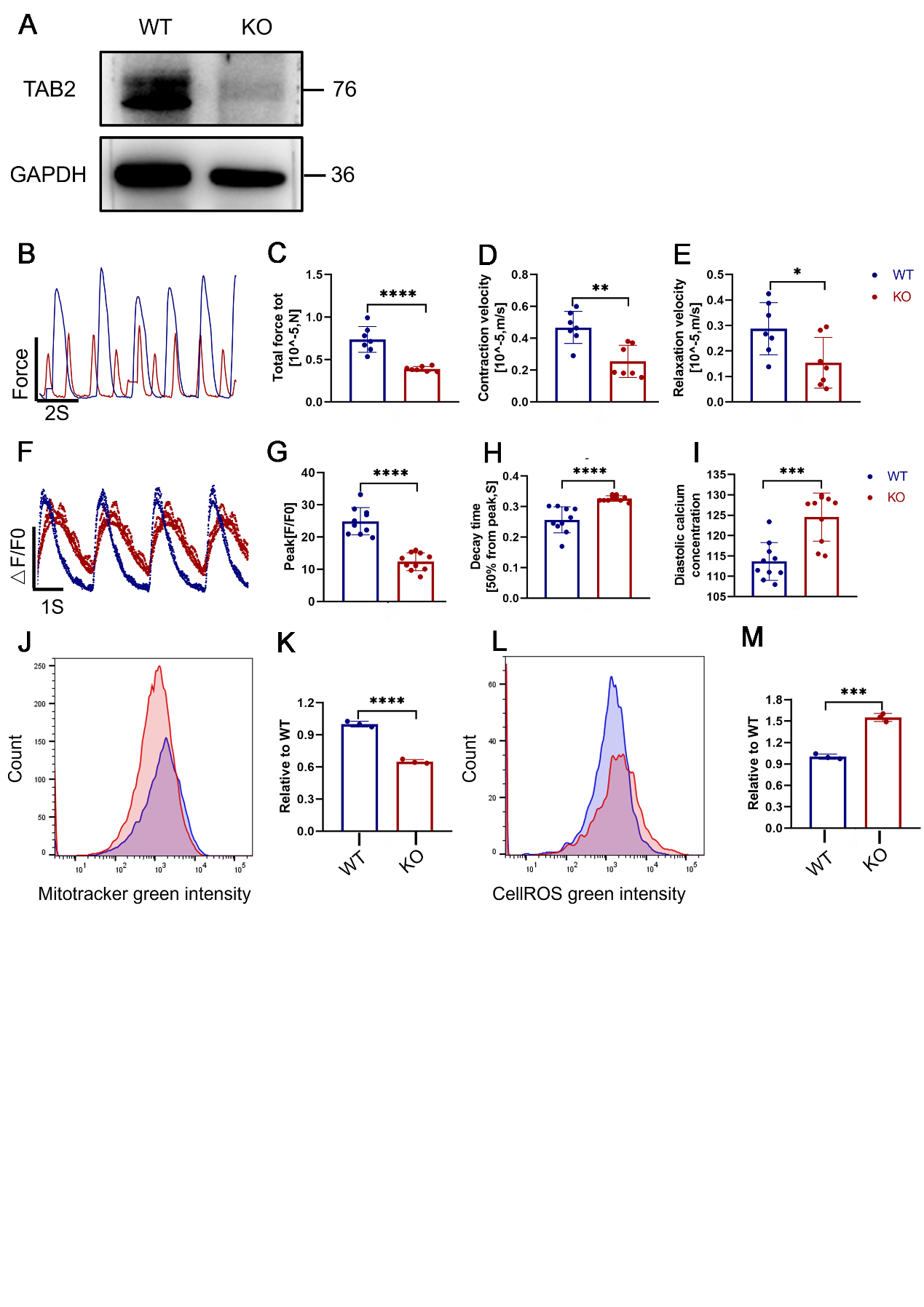


**Supplement Fig 6** Nec-1s rescued the contractility, calcium transients, and mitochondrial content of TAB2-deficient CMs.


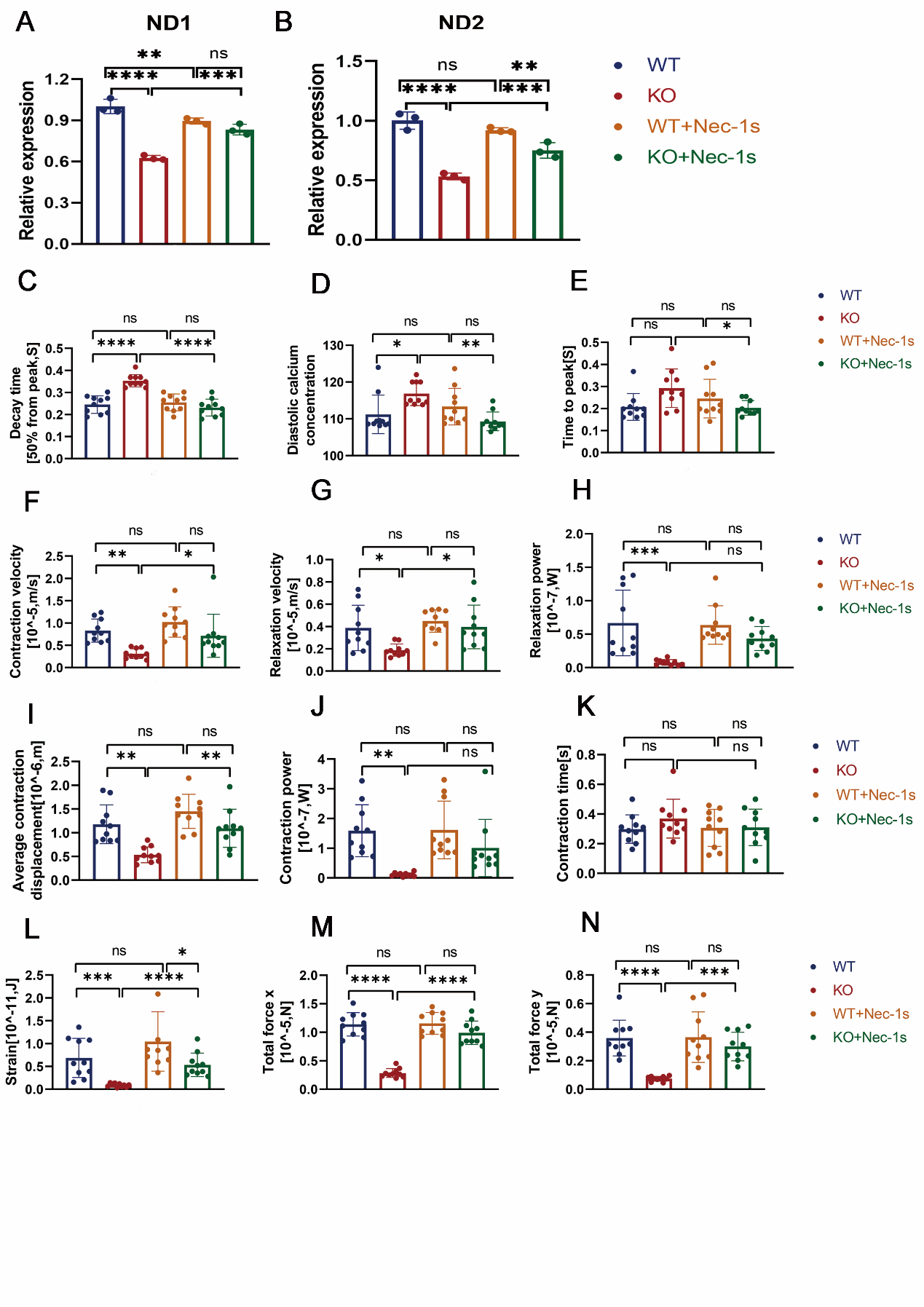


**Supplement Fig 1** The pluripotency of TAB2 knockout iPSCs. **A** Clones morphology in WT and TAB2 konckout induced pluripotent stem cells. **B** Quantification of NANOG, OCT4, SOX2 and REX1 normalized by GAPDH in WT and TAB2 KO hiPSCs (n=9). **C, D** Flow cytometry analysis for SSEA4 from representative WT and TAB2 KO iPSCs(n=3). **E, F** Flow cytometry analysis for TNNT2 from representative WT and TAB2 KO differentiation protocols after purification(n=3).

**Supplement Fig 2** Other contractility data of TAB2 knockout iPSC-CMs. **A-H** Quantification of average contraction displacement, contraction power, contraction time, relaxation power, frequency, strain, total force x and total force y in WT and TAB2-KO hiPSC-CMs (n = 10 cells per group).

**Supplement Fig 3** Comparison of transcriptome sequencing between WT and TAB2 ko cell lines. **A** Schematic demonstrating the GCaMP-expression cassette that was integrated into AAVS1 of WT and TAB2 KO hESCs via nickase CRISPR/Cas9 editing. **B** Principal Component Analysis (PCA) between WT and KO hiPSC-CMs(n=3). **C** Volcano Plot was used to detect differentially expressed genes in WT and KO hiPSC-CMs. **D** GO analysis of pathway enrichment changes in KO hiPSC-CMs. **E, F** Heatmap shows expression changes of genes related to Apoptosis and Necroptosis signaling pathway in WT and KO CMs at days 40.

**Supplement Fig 4** Other phenotypic changes of mitochondria damage in TAB2 deficient cell lines. **A, B** qPCR analysis of mitochondrial DNA (ND1 and ND2) to nuclear DNA(β-actin) ratio at days 30, 40, and 50 of cardiac differentiation (n = 9). **C** Immunostaining of Cell ROS Green in WT and KO hiPSC-CMs at day 40. **D** Quantitative analysis of Cell ROS immunofluorescence intensity (n=5 cells per group). **E, F** Quantification of Rhod, AM red intensity obtained by flow cytometry in TAB2-KO hiPSC-CMs at days 25 as compared with WT hiPSC-CMs(n=3). Results are presented as means ±S.E.M. of three independent experiments. *P < 0.05; **P < 0.01; ***P < 0.001; ****P < 0.0001; ns, not significant, unpaired two-sided Student’s t test.

**Supplement Fig 5** The interference of the TAB2 gene in cardiomyocytes leads to impaired cardiac function. **A** Immunoblot analysis of TAB2 in WT and TAB2 interference CMs at day 20(n=3). **B** Representative line scan images in WT, TAB2 interference hiPSC-CMs myocardial contractility at day 40. **C-E** Quantification of total force tot, relaxation velocity, and contraction velocity in WT and TAB2-interference hiPSC-CMs (n = 7 cells per group). **F** Representative line-scan images in WT-GCaMP and TAB2 interference-GCaMP hiPSC-CMs at day 40.**G-I** Quantification of peak, decay time and calcium diastolic concentration in WT-GCaMP and TAB2 interference-GCaMP hiPSC-CMs (n = 7 cells per group). **J-K** Quantification of Mitotracker Green intensity obtained by flow cytometry in TAB2-interference hiPSC-CMs at day 40 as compared with WT hiPSC-CMs(n=3). **L-M** Quantification of Cell ROS Green intensity obtained by flow cytometry in TAB2-interference hiPSC-CMs at day 40 as compared with WT hiPSC-CMs(n=3)

**Supplement Fig 6** Nec-1s rescued the contractility, calcium transients, and mitochondrial content of TAB2-deficient CMs. **A-B** qPCR analysis of mitochondrial DNA (ND1 and ND2) to nuclear DNA(β-actin) ratio in WT, TAB2 KO, WT + Nec-1s, and TAB2-KO + Nec-1s hiPSC-CMs at day 50 (n = 3).**C-E** Quantification of Decay time, Distolic calcium concentration and Time to peak in WT-GCaMP, TAB2 KO-GCaMP, WT-GCaMP + Nec-1s, and TAB2-KO-GCaMP + Nec-1s hiPSC-CMs at day 50 (n = 10 cells per group).**F-N** Quantification of contraction velocity, relaxation velocity, relaxation power, average contraction displacement, contraction power, contraction time, strain, total force x and total force y in WT, TAB2-KO, WT + Nec-1s, and TAB2-KO + Nec-1s hiPSC CMs at day 50 (n = 10 cells per group). The results are presented as means ± SEMs of 3 independent experiments. *p < 0.05; **p < 0.01; ***p < 0.001; ns, not significant, unpaired 2-sided Student’s t test. One-way ANOVA and least significant difference (LSD) test were used to compare the parameters between groups.

**Supplement file1** TAB2 knockout gRNA off-target site detection by Sanger sequencing, and verification of the absence of random plasmid integration events and mycoplasma infection.
