# Supplementary material for: TAB2 deficiency induces dilated cardiomyopathy by promoting mitochondrial calcium overload in human iPSC-derived cardiomyocytes
Source: Mol Med. 2025 Feb 4;31:42. doi: 10.1186/s10020-025-01103-x (PMC11792723; doi:10.1186/s10020-025-01103-x)

*TAB2* knockout gRNA sequence: GTCTGTCGAGTTGTACCACC **AGG**

**Off target**

| target_seq | PAM | gene name | gene id |
| --- | --- | --- | --- |
| GTCTGTCGAGTTGTACCACC | AGG | *TAB2* | ENSG00000055208 |
| GT**GA**GT**A**GAGT**G**GTACCACC | TGG | *MVP* | ENSG00000013364 |
| G**C**CTGTC**C**A**CC**TGTACCACC | TGG | *TEKT5* | ENSG00000153060 |
| GTCT**TGG**GAGTTG**A**ACCACC | AGG | *ATP8B4* | ENSG00000104043 |

**Off-target site sequencing information**

**1.** ***MVP***

**
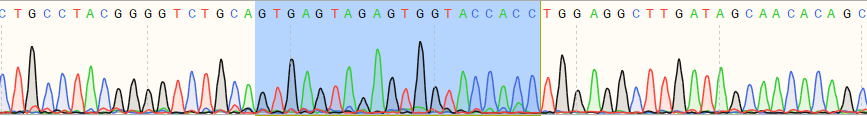
**

**2.** ***TEKT5***

***
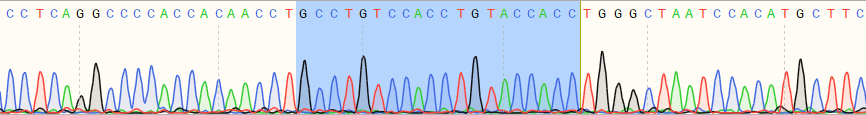
***

**3.** ***ATP8B4***

***
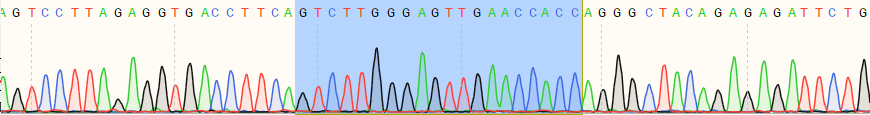
***

***Verification of the absence of random plasmid integration events Verification of the absence of mycoplasma infection***

***
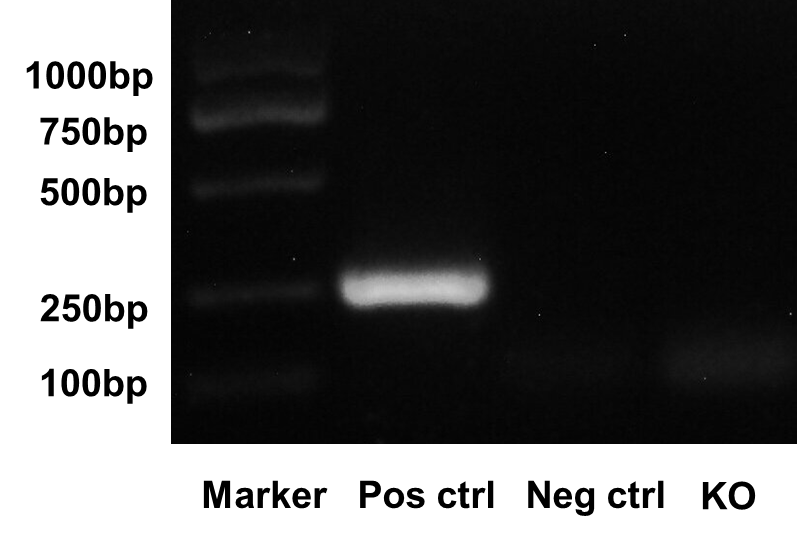
***
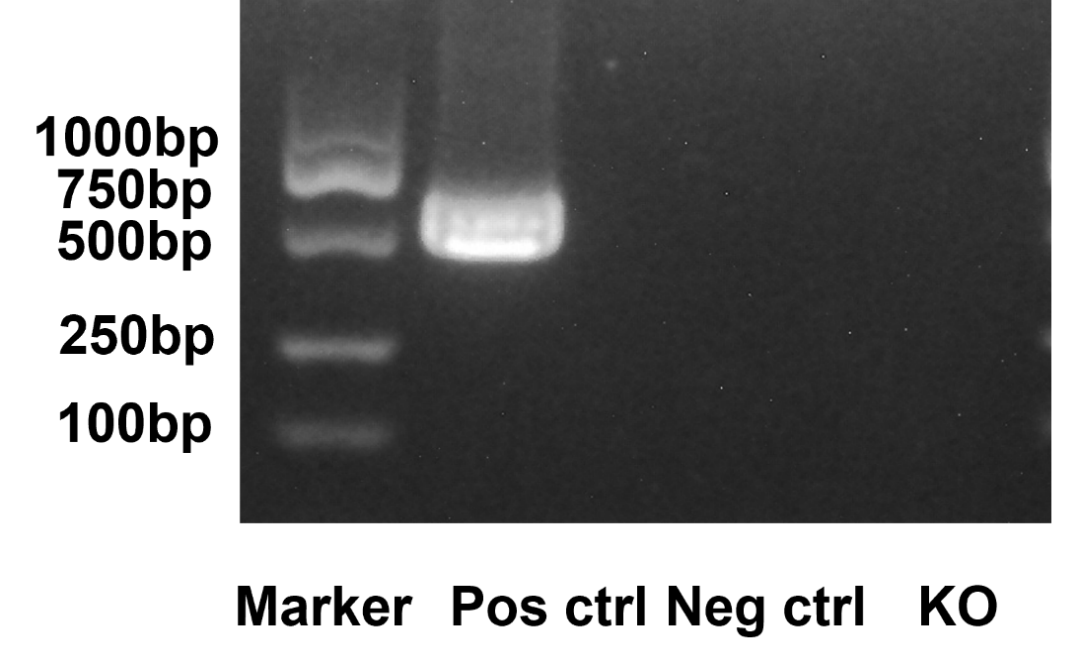

Supplement: Supplementary file 2 — Additional file 2. [file 10020_2025_1103_MOESM2_ESM.docx]
