# Supplementary figures and images for: TAB2 deficiency induces dilated cardiomyopathy by promoting mitochondrial calcium overload in human iPSC-derived cardiomyocytes
Source: Mol Med. 2025 Feb 4;31:42. doi: 10.1186/s10020-025-01103-x (PMC11792723; doi:10.1186/s10020-025-01103-x)

**Related files**

**WB**

**TAB2-KO**




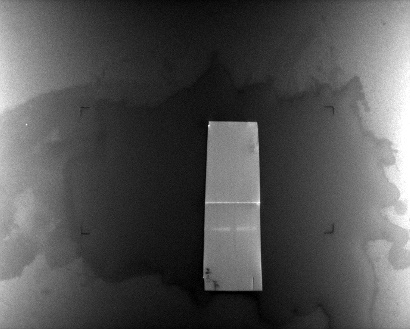


**RIPK1**


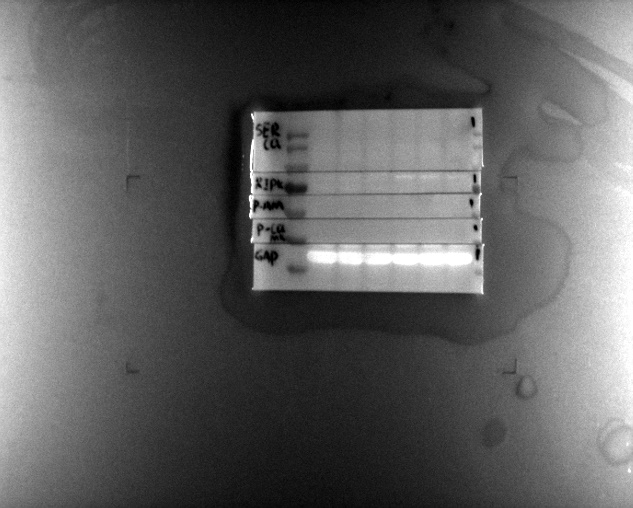

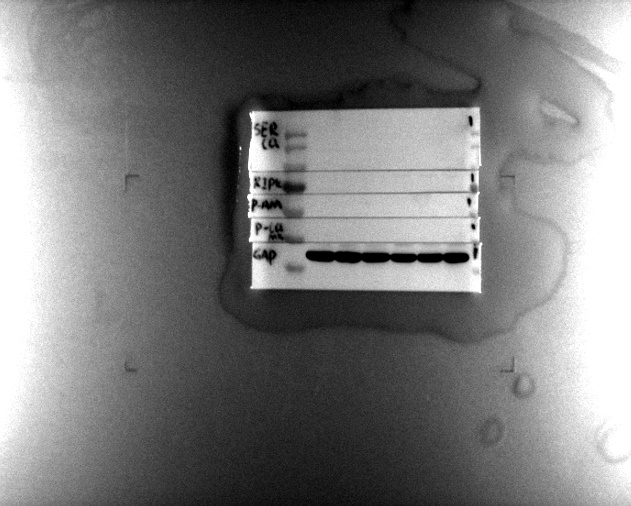


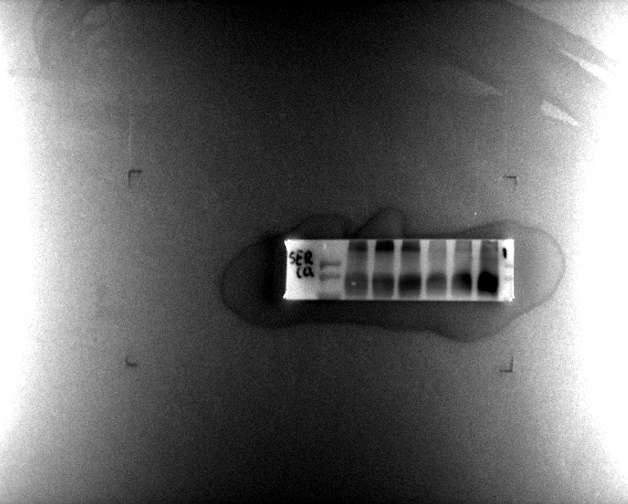


**MCU**


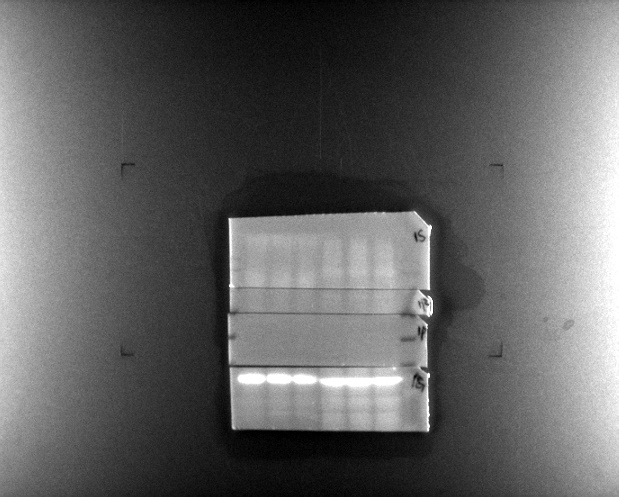

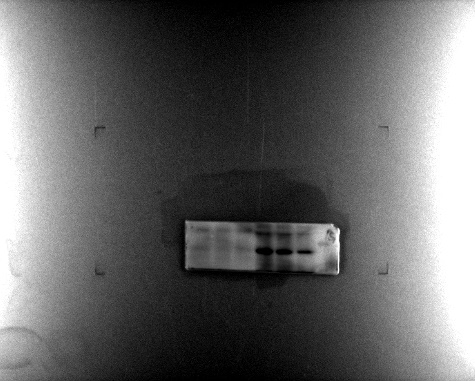


**P-AMPK**


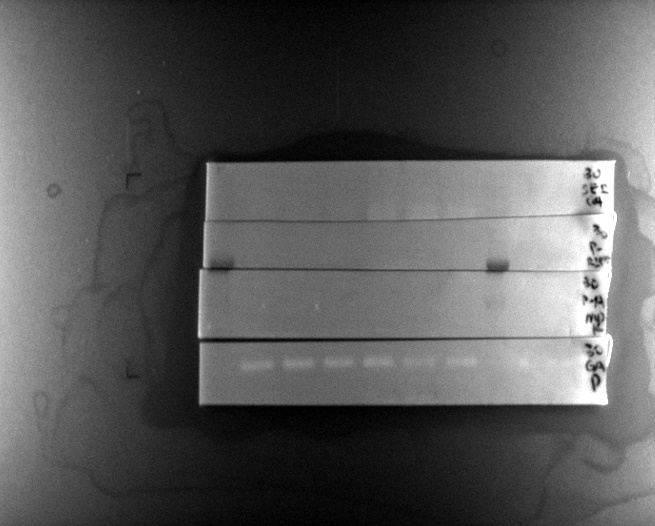




**P-CAMKⅡ**


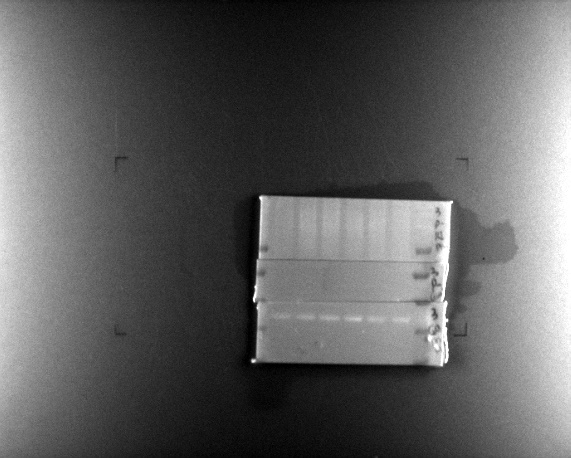



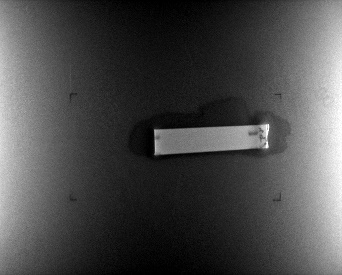


**加药后RIPK1、MCU、P-AMPK**


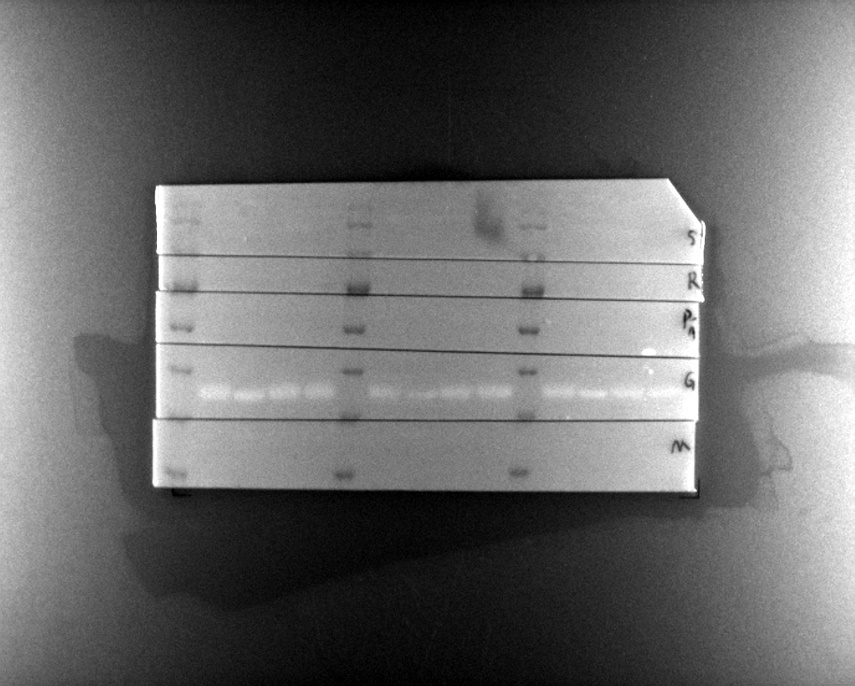


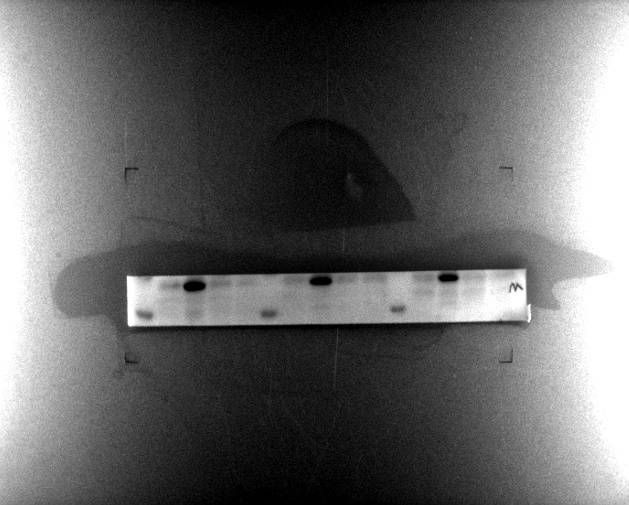

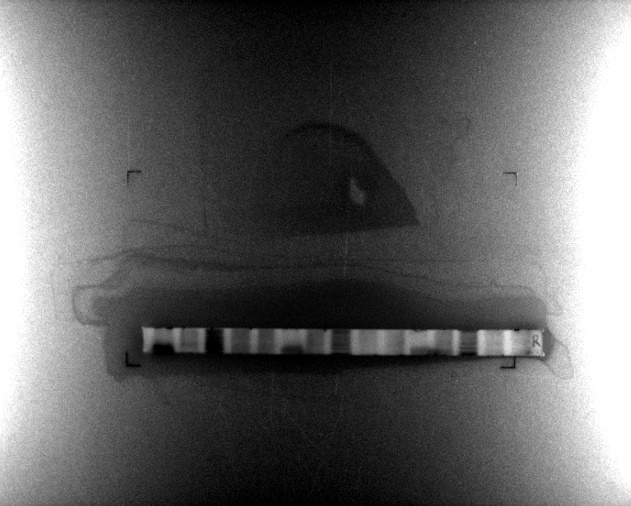


**MCU RIPK1**


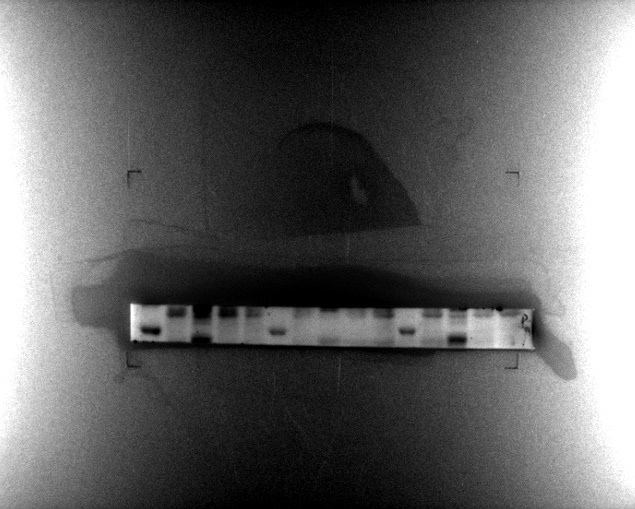


**P-AMPK**

Supplement: Supplementary file 3 — Additional file 3. [file 10020_2025_1103_MOESM3_ESM.docx]
